# Radiopharmaceutical transport in solid tumors via a 3-dimensional image-based spatiotemporal model

**DOI:** 10.1038/s41540-024-00362-4

**Published:** 2024-04-12

**Authors:** Anahita Piranfar, Farshad Moradi Kashkooli, Wenbo Zhan, Ajay Bhandari, Babak Saboury, Arman Rahmim, M. Soltani

**Affiliations:** 1 Department of Mechanical Engineering, K. N. Toosi University of Technology, Tehran, Iran; 2 School of Engineering, King’s College, University of Aberdeen, Aberdeen, AB24 3UE UK; 3 Biofluids Research Lab, Department of Mechanical Engineering, Indian Institute of Technology (Indian School of Mines), Dhanbad, 826004 India; 4 Department of Computational Nuclear Oncology, Institute of Nuclear Medicine, Bethesda, MD USA; 5 Department of Integrative Oncology, BC Cancer Research Institute, Vancouver, BC Canada; 6 Departments of Radiology and Physics, University of British Columbia, Vancouver, BC Canada; 7 Department of Electrical and Computer Engineering, University of Waterloo, Waterloo, ON Canada; 8 Centre for Biotechnology and Bioengineering (CBB), University of Waterloo, Waterloo, ON Canada

**Keywords:** Computer modelling, Computational science, Cancer, Biological physics

## Abstract

Lutetium-177 prostate-specific membrane antigen (^177^Lu-PSMA)-targeted radiopharmaceutical therapy is a clinically approved treatment for patients with metastatic castration-resistant prostate cancer (mCRPC). Even though common practice reluctantly follows “one size fits all” approach, medical community believes there is significant room for deeper understanding and personalization of radiopharmaceutical therapies. To pursue this aim, we present a 3-dimensional spatiotemporal radiopharmaceutical delivery model based on clinical imaging data to simulate pharmacokinetic of ^177^Lu-PSMA within the prostate tumors. The model includes interstitial flow, radiopharmaceutical transport in tissues, receptor cycles, association/dissociation with ligands, synthesis of PSMA receptors, receptor recycling, internalization of radiopharmaceuticals, and degradation of receptors and drugs. The model was studied for a range of values for injection amount (100–1000 nmol), receptor density (10–500 nmol•l^–1^), and recycling rate of receptors (10^–4^ to 10^–1^ min^–1^). Furthermore, injection type, different convection-diffusion-reaction mechanisms, characteristic time scales, and length scales are discussed. The study found that increasing receptor density, ligand amount, and labeled ligands improved radiopharmaceutical uptake in the tumor. A high receptor recycling rate (0.1 min^–1^) increased radiopharmaceutical concentration by promoting repeated binding to tumor cell receptors. Continuous infusion results in higher radiopharmaceutical concentrations within tumors compared to bolus administration. These insights are crucial for advancing targeted therapy for prostate cancer by understanding the mechanism of radiopharmaceutical distribution in tumors. Furthermore, measures of characteristic length and advection time scale were computed. The presented spatiotemporal tumor transport model can analyze different physiological parameters affecting ^177^Lu-PSMA delivery.

## Introduction

Therapeutic radiopharmaceuticals represent agents that combine the specificity of a targeting molecule with the therapeutic effect of a radioisotope, enabling precise and targeted cancer treatment^1^. Among these, lutetium-177 prostate-specific membrane antigen (^177^Lu-PSMA) targeted therapy has emerged as a promising approach for treating advanced prostate cancer^2^. PSMA is highly expressed on the surface of prostate cancer cells, making it an ideal target for therapy. Lutetium-177, a beta-emitting radionuclide, emits high-energy beta particles that can effectively damage cancer cells^3,4^. The binding of ^177^Lu-PSMA to PSMA-expressing cells enables the localized delivery of radiation, resulting in targeted cell destruction and tumor regression^5^. The limitations of conventional, one-size-fits-all treatments become apparent when confronted with the intricate complexities and heterogeneity of prostate cancer^6,7^. These challenges necessitate a shift towards personalized treatment approaches. ^177^Lu-PSMA therapy, emblematic of this paradigm shift, seeks to tailor treatment strategies based on each patient’s unique disease characteristics, a concept strongly supported by both clinical evidence and a compelling conceptual argument^8^. In the realm of clinical applications, ^177^Lu-PSMA has traditionally been tailored for the treatment of metastatic castration-resistant prostate cancer (mCRPC)^9–12^. However, an emerging paradigm envisions its deployment in the nascent phases of prostate cancer, preceding metastasis, and any therapeutic interventions such as radiation or prostatectomy^13,14^. Treatments with ^177^Lu-PSMA have demonstrated impressive responses, including tumor size reduction and decreased levels of prostate-specific antigen (PSA, which is a key biomarker for prostate cancer)^15,16^. Additionally, ^177^Lu-PSMA therapy has shown promise in extending overall survival and delaying disease progression in prostate cancer patients^17,18^.

Pharmacokinetic models assess the distribution and elimination of ^177^Lu-PSMA within the body, while dosimetry models estimate the absorbed radiation dose delivered to tumors and healthy tissues^19^. Biokinetic models delve deeper into the radiobiological effects of therapy, considering cellular processes and DNA damage. Physiologically based pharmacokinetic (PBPK) modeling is a computational approach used to describe absorption, distribution, metabolism, and excretion, allowing to predict drug concentrations in various tissues over time^10^. PBPK models, though providing a holistic kinetics of the drug in the body, do not focus specifically on the spatiotemporal aspects of radiopharmaceutical distributions within tumors and organs. The studies that concentrate exclusively on PBPK models to investigate ^177^Lu-PSMA delivery^9,10,16,20^ do not account for complexities of tumor microenvironment and its influence on interstitial kinetic.

The emergence of spatiotemporal distribution models (SDMs) has enhanced our understanding of the spatial and temporal dynamics of the radiopharmaceutical agents. In the computational modeling of drug delivery systems, SDMs have been shown value owing to their ability to assess solute transport through convection, diffusion, and reaction phenomena^21^. Microvascular conductivity, transvascular permeability, and interstitial space aspects are only a few examples of the fundamental physiological properties that may be fully accounted for by SDMs^22,23^.

Medical images offer valuable data sources for the development of the models. Recent computational models designed to predict drug distribution in the body have created new opportunities for personalized treatment by adopting personal data interpreted from medical imaging data. For instance, Bhandari et al.^24,25^ developed an image-based 3-dimensional computational fluid dynamics (CFD) model for investigating drug delivery to human brain tumors, in which the patient-specificity was incorporated using dynamic contrast-enhanced magnetic resonance imaging (DCE-MRI) data. DCE-MRI data provides valuable insights into the tissue’s permeability to the contrast agent, the interstitial volume fraction of the tissue, and the patient-specific arterial input function, offering practical and useful information^25^. An image of the tumor was used by Moradi Kashkooli et al. to evaluate nanoparticle delivery and predict treatment efficacy^26^. Several other studies have also used the same imaging modality to investigate drug transport in murine tumors, including Magdoom et al.^27^ and Pishko et al.^28^. Few studies have designed 3-dimensional drug delivery models based on realistic data of tumors. Zhan et al.^29^ developed a 3-dimensional computational model to investigate doxorubicin (DOX) delivery in solid tumors, and the geometry of the tumor was reconstructed from MR images. Researchers established a 3-dimensional realistic brain tumor model based on convection-diffusion-reaction (CDR) equations and Darcy’s law in another study^30^. Caddy et al.^31^ developed a 3-dimensional CDR model using a computerized tomography image to study the transport of nanoparticles within the tumor. Models based on medical images, when combined with SDMs, contribute to a deeper understanding of ^177^Lu-PSMA behavior and can be used to plan and optimize individual treatment regimens.

Due to the limited existing literature, there is a need for further research to adequately study the distribution of ^177^Lu-PSMA in solid tumors. By addressing this knowledge gap in radiopharmaceuticals delivery, more realistic and effective treatment choices can be made. Motivated by this, the current study, for the first time to our knowledge, implements 3-dimensional numerical modeling to analyze ^177^Lu-PSMA-617 kinetic in prostate tumors. In this study, particular attention is given to PSMA-617, a radioligand of significant relevance^6^. PSMA-617, characterized as a small molecule, harbors distinctive features that render it an optimal candidate for therapeutic application^32^. Its notable attribute lies in the remarkable affinity it demonstrates for PSMA, enabling the selective binding to this cell surface protein expressed on prostate cancer cells^17^. This precise targeting mechanism ensures that the therapeutic intervention is predominantly directed towards malignant cells, thereby curbing unintended damage to healthy tissues^6^. Additionally, PSMA-617 possesses the capacity for internalization by prostate cancer cells. Following its initial binding to PSMA on the cellular surface, the radioligand is internalized, consequently enhancing the treatment’s overall efficacy^6^. In this regard, the 3-dimensional geometry of tumors and surrounding healthy tissue was reconstructed from MR images. The impact of receptor density, the recycling rate of PSMA receptors, injection amount, and continuous injection on the concentration of ^177^Lu-PSMA-617 in normal and tumor cells were investigated in detail.

## Results

### Spatiotemporal changes in the concentration of ^177^Lu-PSMA-617

**Fig. 1:**
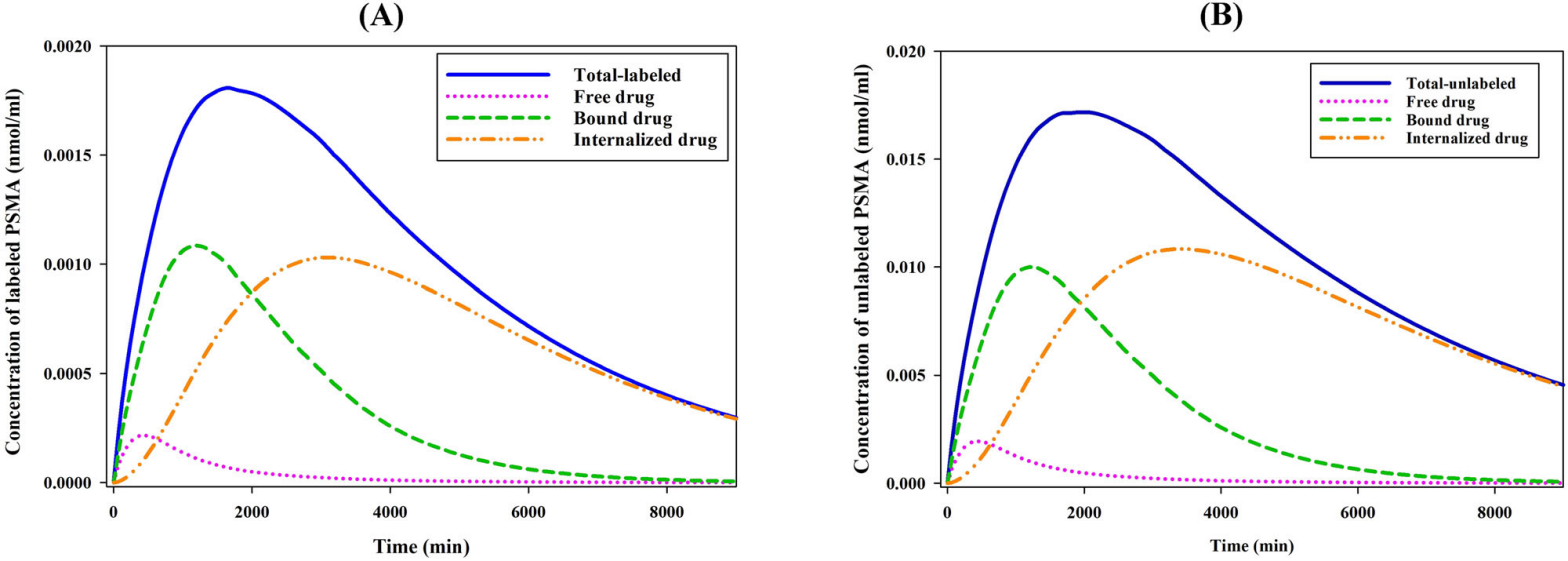
Temporal distribution of PSMA-617 pharmaceuticals in tumor. The distribution of (**A**) labeled and (**B**) unlabeled free (C_F_), bound (C_B_), internalized (C_I_), and total PSMA-617 pharmaceutical concentrations over time in the tumor.

Figure 1 illustrates the concentration of ^177^Lu-PSMA-617 in distinct compartments within tumor 1. When the free radiopharmaceutical enters the interstitial space, it binds to receptors on the cell surface. Radiopharmaceuticals initially bind to receptors rapidly, but as receptors are saturated, fewer radiopharmaceuticals can attach to the cell membrane. Next, the PSMA ligand-protein complex is internalized into the tumor cells via clathrin-mediated endocytosis. Since the β-rays of ^177^Lu have an average soft tissue range of 0.23 mm (surpassing the cell diameter), internalization of ^177^Lu-PSMA-617 is unnecessary^7^. Finally, radiopharmaceuticals degrade or are released. The concentrations of bound and free radiopharmaceuticals reduce to an undetectable level. Considering that about 90% of the injected drug is the unlabeled ligand, its concentration in the tissue is higher than that of the labeled one.

Supplementary Figure 1 illustrates the spatiotemporal variations of the labeled PSMA-617 concentrations within a tumor 1. First, ^177^Lu-PSMA-617 is transmitted to the interstitial space through convection and diffusion mechanisms. C_B_ and C_F_ are dominant in the early time steps. In addition, the lymphatic vessels remove some of the free radiopharmaceuticals from the interstitial space. However, C_B_ and C_I_ increase gradually due to the free radiopharmaceutical converting to the bound and then internalized ^177^Lu-PSMA-617 by binding and internalizing, respectively. Importantly, free and bound radiopharmaceutical concentration changes are in opposite directions. Furthermore, the concentration of radiopharmaceuticals in the tumor region surpasses that of normal tissue in the contours depicting bound, internalized, and overall concentrations (Supplementary Fig. 1). The maximum value of the total concentration occurs in the tumor region. This value is several times higher than the concentration in the normal tissue around it.

In the Supplementary Material file, as demonstrated in Supplementary Fig. 2 (for cut planes 1–4) and Fig. 1 (for cut plane 5), a comprehensive comparison of spatial changes in radiopharmaceutical concentration in different Cut plane were provided. In this study, the selection of cut planes was carefully made to encompass regions of interest within the tumor as well as the adjacent normal tissue (Fig. 3). The radiopharmaceutical is uniformly distributed across all cut lines in the geometry of Tumor 1 and its surrounding tissue (Supplementary Fig. 2). It is noteworthy that the consistently highest concentrations manifest along the interface between the tumor and normal tissue. However, an intriguing observation emerges specifically within cut plane 2, where, at varying time intervals, the drug concentration exhibits a more pronounced localization in regions characterized by a narrower tumor geometry. Furthermore, the analysis consistently underscores that the concentration of radiopharmaceutical within various regions of Tumor 1 consistently exceeds that observed in the adjacent normal tissue.

Supplementary Figure 3 illustrates the temporal evolution of radiopharmaceutical concentration in distinct XY plane cut lines. The findings reveal notable variations in concentration levels among the cut lines. Specifically, Cutline 2 exhibits the highest concentration, primarily attributed to its substantial tumor cell content compared to normal cells. Conversely, Cutline 3, situated within the normal tissue region of Tumor 1 geometry, demonstrates the lowest radiopharmaceutical concentration. Supplementary Figure 4 illustrates the spatial distribution of radiopharmaceutical concentration along Cutline 2. Throughout all time points, the tumor consistently maintains a higher concentration of the radiopharmaceutical than the surrounding normal tissue, with the greatest concentration observed at the tumor periphery. This phenomenon is attributed to elevated interstitial fluid pressure within the tumor core, which drives the preferential drug distribution toward the tumor’s outer regions.

### Effects of receptor density, its recycling rate, and injection amount on concentration

**Fig. 2:**
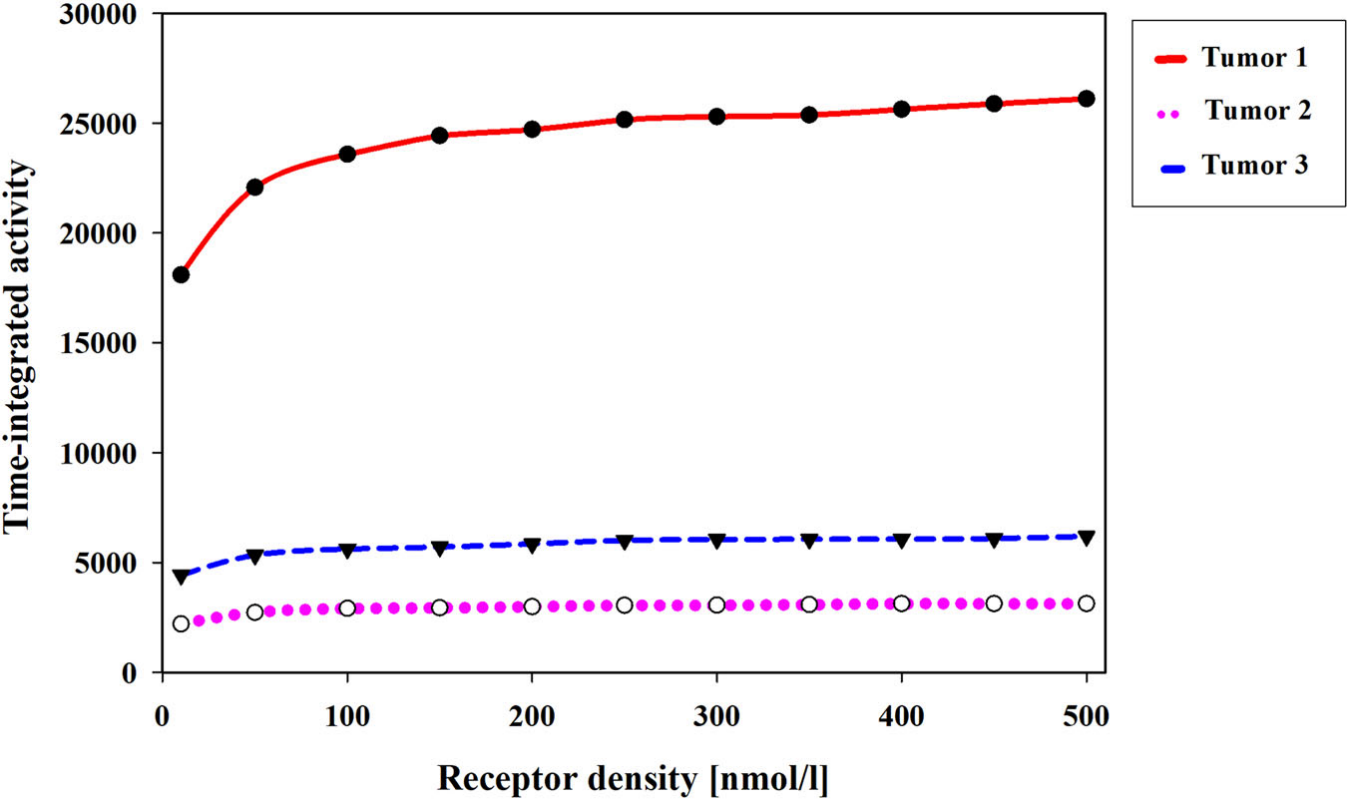
Effects of receptor density (R_d) on TIA (MBq.min) in the tumor. Increased receptor density increases radiopharmaceutical TIA in tumors.

Based on the size and volume of the modeled tumors, the PSMA receptor density ranges between 10 and 500 nmol/L^10^, and an increase in receptor density results in a corresponding rise in the time-integrated activity (TIA, MBq.min) within tumors, as depicted in Fig. 2. TIA represents the cumulative activity of a radiopharmaceutical in a given region over time, and it is an essential parameter for assessing the total radiation dose delivered by radiopharmaceuticals in a dynamic system. Mathematically, TIA can be defined as the integral of the total concentration of a radiopharmaceutical concerning time.

In the case of Tumor 1, the results demonstrate a significant and rapid increase in the concentration of radiopharmaceuticals as the receptor density rises from 10 to 100 nmol/L. This phenomenon can be attributed to a larger surface area of the tumor being exposed to the radiopharmaceuticals, thereby facilitating enhanced radiopharmaceutical uptake. However, beyond a receptor density of 100 nmol/L, although the TIA continues to increase, the slope of the TIA graph starts to decrease. As a result, further increments in receptor density may not lead to proportionate increases in radiopharmaceutical concentration within the tumor. This pattern is also observed for the other two tumors, as illustrated in Fig. 2.

**Fig. 3:**
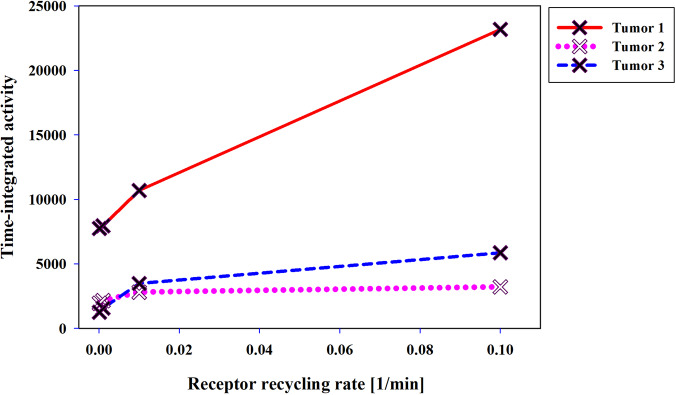
Effects of the receptor recycling rate (k_rec) on TIA in tumors. A recycling rate constant (k_rec) of 0.1 min^–1^ results in a radiopharmaceutical concentration surpassing that of when the recycling rate constant is 0.01 and 0.001 min^–1^.

The next parameter investigated is the recycling rate of PSMA receptors. In the realm of cellular processes, the recycling of PSMA receptors assumes a pivotal role, dynamically regulating receptor abundance on the cell surface for subsequent ligand binding and signaling events^33,34^. This intricacy is of utmost importance for radiopharmaceutical distribution and effectiveness within the tumor microenvironment. Interestingly, the recycling rate of PSMA receptors exhibits sensitivity to both the receptor count and the dimensions of the ligands^34^. Consequently, the recycling rate decreases accordingly in response to these factors. Such insights into the recycling dynamics of PSMA receptors hold great promise for optimizing drug-targeting strategies and advancing therapeutic interventions for various malignancies, including prostate cancer^35^. Based on Fig. 3, K_rec_ = 0.1 min^–1^ results in the highest TIA value. There is almost no difference in the TIA for recycling rates between 0.0001 and 0.001. The elevated recycling rate facilitates a more rapid turnover of receptors, creating a favorable environment for the enhanced binding of labeled peptides in the tumor, ultimately leading to an increase in their concentration. This finding highlights the importance of receptor recycling kinetics in modulating the concentration of labeled peptides.

Based on the results, increasing the recycling rate from 0.0001 to 0.1 min^–1^ led to substantial enhancements in the TIA. The TIA in the first, second, and third tumors experienced approximately 3, 1.6, and 4 fold increases, respectively. Notably, the second tumor, characterized by the highest cell receptor density among the three tumors, exhibited a relatively smaller increase in the TIA compared to the other tumors when the recycling rate was elevated. This observation highlights the intricate interplay between receptor density and the recycling rate, underscoring their combined influence on drug distribution. The findings underscore the crucial role of receptor density in the significant impact of the recycling rate on drug uptake and accumulation within the tumor microenvironment.

**Fig. 4:**
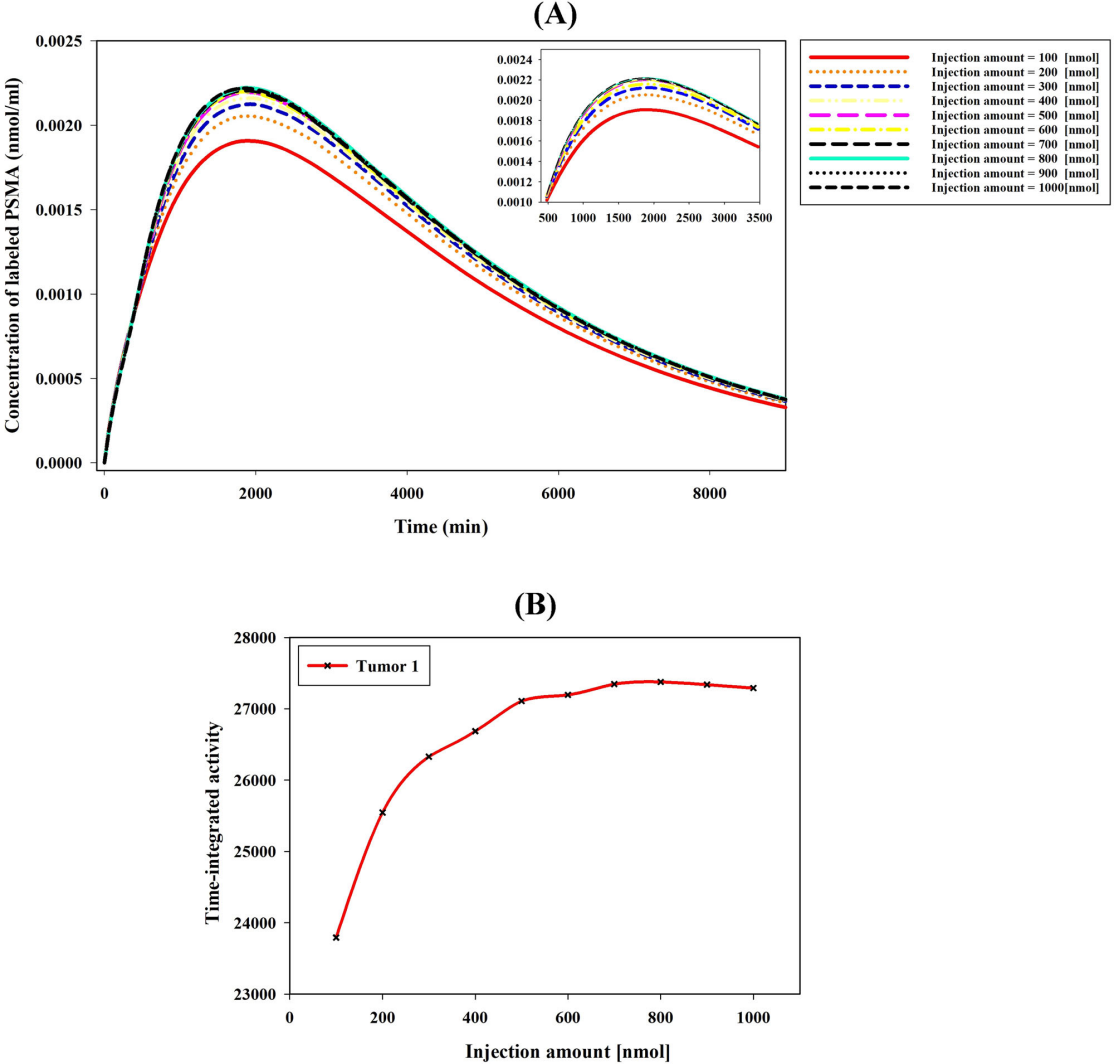
Impact of radiopharmaceutical amount on PSMA-617 concentration. **A** Investigating the effects of the administered radiopharmaceutical amount on the labeled PSMA-617 concentration in the tumor. The injected radiopharmaceutical comprises 10% labeled ligands and 90% unlabeled ligands. **B** Raising the injection amount of labeled and unlabeled ligands initially boosts TIA, but at high injections, receptor saturation by unlabeled ligands leads to decreased TIA.

The study examines the impact of pharmaceutical injection amounts on concentrations ranging from 100 to 1000 nmol, comprising 10% labeled peptides and 90% unlabeled peptides. Consequently, as the injected pharmaceutical amount increases, both labeled and unlabeled ligands exhibit a corresponding increase. Figure 4 illustrates the TIA in tumor 1, which demonstrates an increase with an escalating injection amount ranging from 100 to 800 nmol. However, this excessive increase is not significant since the number of unlabeled peptides increases more than that of labeled peptides with an increase in total peptides. Furthermore, a reduction in radiopharmaceutical concentration and TIA within the tumor is observed when the injection amount exceeds 800 nmol. This phenomenon occurs due to the saturation of cell surface receptors caused by binding to unlabeled ligands. Consequently, cell surface receptors, which have fixed capacities, become overwhelmed by a substantially higher quantity of unlabeled peptides, thereby reducing the availability of binding sites for labeled PSMA. Referring to Supplementary Fig. 5, it is evident that as injection amounts increase, tumor 2 and tumor 3 display similar patterns to tumor 1.

**Fig. 5:**
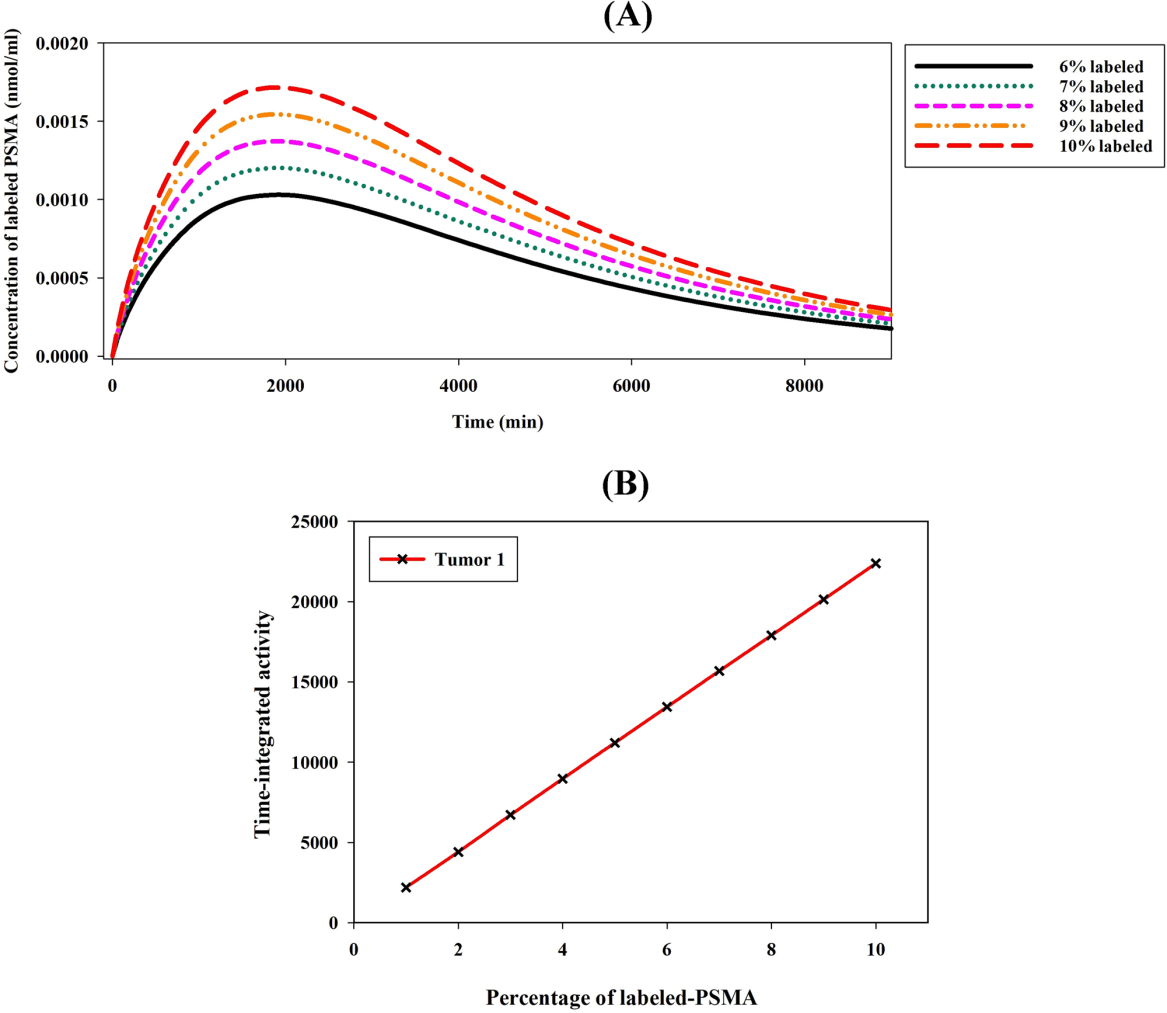
Effect of labeled PSMA-617 percentage on total concentration. **A** Investigating the influence of the percentage of labeled PSMA-617 on the total concentration of labeled PSMA in the tumor. **B** The results demonstrate a significant increase in TIA as the percentage is elevated from 1 to 10%.

Additionally, the study investigates the impact of the labeled PSMA amount on TIA while keeping the total injection amount constant at 100 nmol. The results demonstrate that as the percentage of labeled PSMA is increased from 1 to 10%, the TIA consistently rises (Fig. 5). Across all tumors, the TIA increases approximately 10 times with the elevation of labeled PSMA percentage from 1 to 10% (Supplementary Fig. 6).

### Continuous infusions

**Fig. 6:**
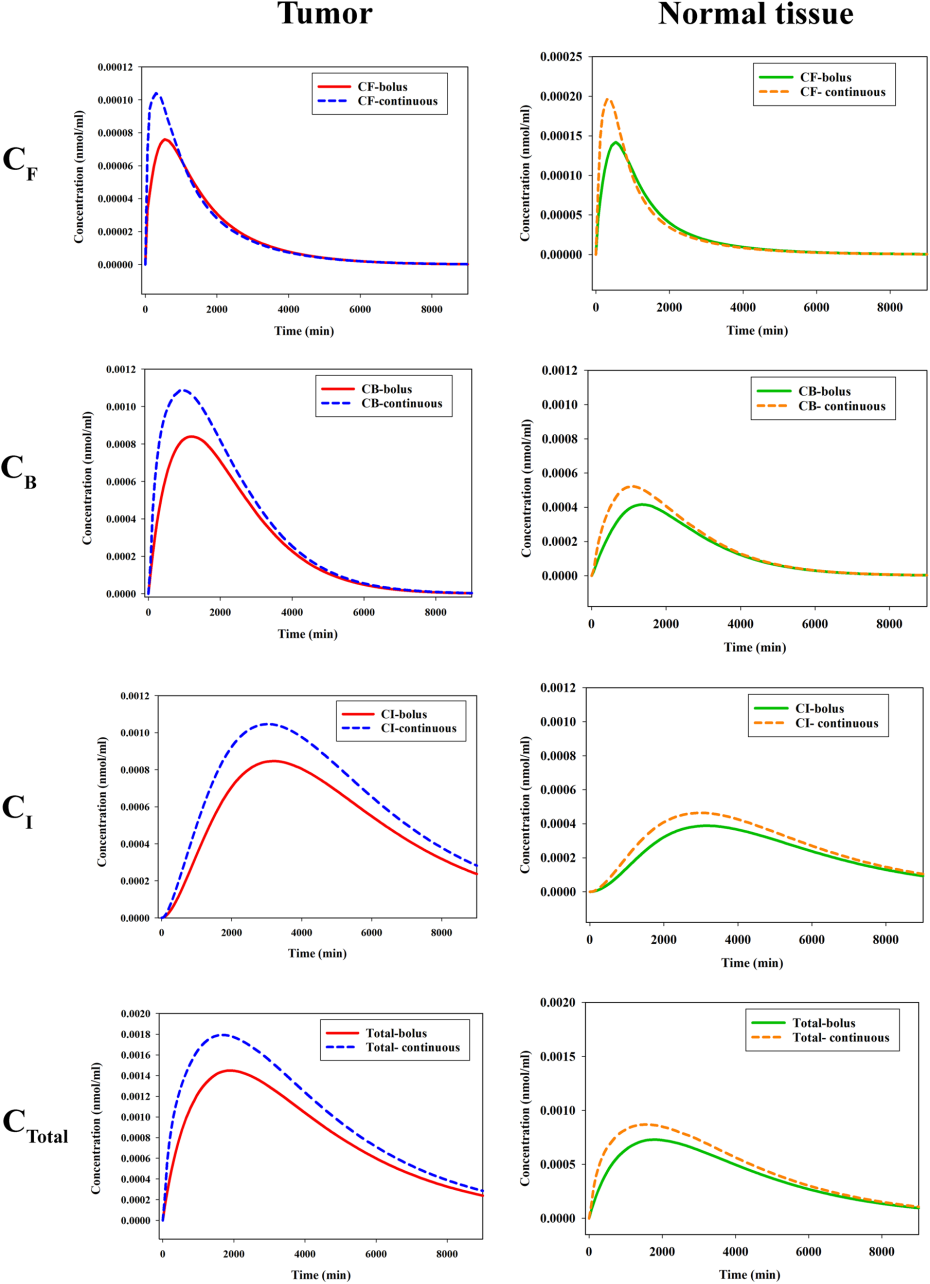
Bolus vs. continuous infusion. Plots of free (C_F_), bound (C_B_), internalized (C_I_), and total (C_T_) concentrations of the labeled PSMA-617. The figure illustrates the concentrations of the labeled PSMA-617 in the tumor (left column) and normal tissue (right column) using 2 different administration methods: bolus injection and continuous infusion.

In this study, the comparison between bolus injection and continuous infusion of ^177^Lu-PSMA-617 was investigated to determine the concentration of the labeled PSMA-targeted ligand in both tumor and normal cells. The results, as depicted in Fig. 6, highlight distinct concentration patterns in various spaces: extracellular, receptor-bound, and intracellular. Notably, continuous infusion yielded different outcomes compared to bolus injection. The prolonged delivery of radiopharmaceutical over 60 min led to a higher peak concentration of free radiopharmaceutical in the extracellular space, achieving more gradual clearance through vessels in comparison to bolus injection. Additionally, the continuous infusion approach exhibited a more pronounced peak concentration of radiopharmaceuticals that were bound to receptors in comparison to the bolus administration method. This outcome can be attributed to the phenomenon of receptor saturation manifesting with reduced intensity during continuous infusion. The extended timeframe of continuous injection allows receptors to repopulate on cell surfaces, affording a conducive environment for heightened binding of radiopharmaceuticals. Consequently, this intensified binding contributes to an augmentation in the concentration of radiopharmaceuticals within the intracellular compartment. Remarkably, the total concentration of labeled PSMA are observed to increase with continuous infusion compared to bolus injection. Specifically, TIA values were lower for bolus injection compared to continuous infusion, underscoring the potential advantages of the latter approach in enhancing radiopharmaceutical delivery and retention within the tumor microenvironment.

### Non-dimensional analysis of time and length scales

Non-dimensional analysis simplifies radiopharmaceutical delivery by normalizing variables and revealing fundamental relationships and dominant factors. The free and bound radiopharmaceuticals as functions of the distance from the blood vessel wall located at x = 0 were analyzed. A nonzero but constant interstitial fluid velocity (IFV) is assumed, advecting the material away from (*v* > 0) or toward the vessel wall (*v* < 0). Moreover, it is assumed that vascular concentration changes gradually, allowing for the development of nearly steady-state conditions near the blood vessel walls. The distance from the vessel wall would exponentially reduce ligand penetration from tumor blood vessels without advection. In this case, based on Eq. (5), for example, when $D$ = 8.7 × 10^−7^ cm2 s^−1^ ^36^ and ${K}_{F}$ = 0.038 s^–1^ ^10^, the characteristic length scale equals L_F_ ≈ 4.7 × 10^−4^ cm for delivery it. As IFV is in the order of 10^–8^ m.s^–1^, the characteristic time scale is also 4.7 × 10^3^ s. Hence, from Eq. (6), the characteristic diffusion time scale can be calculated as 2.5 × 10^1^ s. Finding the Peclet number before calculating the advection time scale is necessary. The Peclet number equation can be written as Pe$=\frac{{L}_{P}(\bar{{P}_{B}}-\bar{{P}_{i}}-{\sigma }_{s}({\pi }_{B}-{\pi }_{i}))\left(1-{\sigma }_{f}\right)}{{P}_{n}}$. ^37^ By implementing the average blood pressure ($\bar{{P}_{B}}=$2,079.829 Pa) and the average interstitial fluid pressure (IFP) ($\bar{{P}_{i}}=$ 1500 Pa), the Peclet number yields approximately 3 × 10^–3^. Since the advection time scale is the multiple of the Peclet number and the characteristic diffusion time scale (T_a_= T_d_ /*Pe*), the advection time scale would be 8.3 × 10^1^ s.

As a result, longer characteristic lengths are generally associated with a more uniform distribution of the radiopharmaceutical, but at the cost of potentially lower concentrations. The radiopharmaceutical must also have a low binding affinity for deep penetration into the tumor. This is consistent with the findings reported in several studies^38–40^.

## Discussion

^177^Lu-PSMA has been specifically designed for the treatment of mCRPC^9–11^. Recent clinical studies have expanded its application to the early phases of prostate cancer, occurring prior to metastasis and therapeutic interventions^13,14^. The promising insights gleaned from preliminary results, notably from studies such as the LuTectomy Trials^13^, underscore the imperative for further investigation and the incorporation of this evolving landscape into the ongoing discourse on prostate cancer treatment strategies. In this study, the utilization of SDMs facilitates the analysis of various parameters related to radiopharmaceutical transport within prostate tumors, including the density of receptors, recycling rate of PSMA receptors, injection amount, and bolus and continuous injection of the radiopharmaceutical^36^. These parameters can be incorporated into the model to assess their influence on the spatiotemporal distribution of the radiopharmaceutical within the tumor microenvironment. As such, they can provide valuable insights into the dynamics of this specific type of prostate cancer treatment.

The current study demonstrates that radiolabeled and unlabeled ligands display the same behavior within tumors over time regarding changes in free, bound, and internalized drug concentrations. However, due to a higher injection volume (90% of the total injection amount), the unlabeled PSMA-617 achieves a greater tumor concentration. In fact, the concentration of unlabeled PSMA-617 is approximately an order of magnitude higher than the labeled counterpart. This finding elucidates that despite the higher concentration of unlabeled ligands within the tumor, it concurrently serves to minimize the risk of detrimental effects on the surrounding normal tissues. This result is in good agreement with the experimental results by Cui et al.^41^.

When considering the computational domain, 2 types of tissues are taken into account: tumors and normal tissues. Notably, the density of PSMA receptors is higher in the tumor area than in normal tissue^9^. This disparity is visually evident in Supplementary Fig. 1. This disparity in radiopharmaceutical concentration enables the success of targeted treatments, making the use of ligands such as PSMA-617 highly promising for improving therapeutic outcomes. This is a well-established topic in the literature^5,42,43^.

Receptor density plays a key role in determining the binding kinetics and absorption of radiopharmaceuticals^9^, meaning that when receptor density increases from 10 to 100 nmol/L, the AUC of the total concentration increases. In tumors, higher receptor density increases initial binding, leading to higher radiopharmaceutical concentrations (Fig. 2). Conversely, lower receptor density may restrict radiopharmaceutical binding and reduce drug accumulation. This finding is supported by previous studies^44,45^. Furthermore, the findings of this study highlight that maintaining a constant injection amount while increasing the receptor density on the cell surface within a specific range substantially contributes to enhancing radiopharmaceutical accumulation in the tumor. However, beyond this particular interval of receptor density, the impact of further increasing the receptor density diminishes. In other words, there exists an optimal range of receptor density that maximizes drug uptake, and exceeding this range does not yield additional benefits in terms of radiopharmaceutical accumulation within the tumor microenvironment. These observations emphasize the significance of receptor density as a key factor in drug-cell interactions.

The recycling rate of receptors is another effective factor in radiopharmaceutical therapy. By increasing the recycling rate of receptors, more receptors are available on the cell surface for radiopharmaceutical binding, leading to enhanced drug uptake. In other words, receptors that undergo rapid recycling have a higher chance of binding to the radiopharmaceutical multiple times, leading to enhanced drug retention and accumulation in the tumor^9,46^. On the other hand, recycling rates higher than 0.01 (min^−1^) can increase radiopharmaceutical binding and reduce the likelihood of drug clearance from the tumor microenvironment (Fig. 3). Understanding the impact of receptor recycling on radiopharmaceutical distribution assist in elucidating the mechanisms underlying drug-cell interactions.

Enhancing the amount of labeled PSMA injected can result in higher initial radiopharmaceutical concentrations in the tumor, which may improve tumor targeting and therapeutic efficacy. By assuming a constant injection amount and increasing the labeled PSMA percentage from 1 to 10%, it is possible to increase the concentration of the radiopharmaceutical in the tumor by more than 10 times (Fig. 5). Higher activity administered enhances the binding of labeled PSMA to tumor cells, thereby enhancing the internalization and retention of the radiopharmaceutical in the cellular space. This potentially results in an increase in radiation dose deposition within the tumor, thereby augmenting the cytotoxic effects on tumor cells^47^.

This investigation highlights the effect of simultaneously increasing unlabeled and labeled PSMA ligands in the context of targeted drug delivery. The findings of the current study demonstrate that the concurrent rise in the abundance of both ligand types leads to competitive interactions. Unlabeled PSMA ligands effectively occupy binding sites on tumor cells that would otherwise be targeted by labeled PSMA ligands. Consequently, despite escalating injected amount, the presence of these competitors only marginally augments the concentration of labeled ligands within tumors (Fig. 4). Moreover, the augmentation of unlabeled ligands beyond a certain threshold value leads to reduced uptake of labeled ligands and the saturation of cell surface receptors with unlabeled ligands. This threshold value varies depending on tumor characteristics, such as the volum of tumor and the receptor density in patients. However, through controlled elevation of the concentration of unlabeled PSMA ligands, it becomes possible to regulate the uptake of labeled ligands in non-target tissues, thereby improving their specificity for tumor cells. Moreover, the supplementary simulations, comparing the administration of a defined dose of pure labeled PSMA (10 nmol) with concurrent dosing of the same quantity along with additional unlabeled PSMA (100 nmol), reveal a noteworthy reduction in TIA from 22978.7 to 17221.4. This phenomenon holds the potential to yield heightened tumor-to-background ratios and mitigate off-target effects, as evidenced in the relevant scientific literature^47,48^.

Lastly, the findings of this study underscore the discernible differences between bolus and continuous infusion techniques concerning the distribution of radiopharmaceuticals within the intricate milieu of the tumor microenvironment. Continuous infusion stands as a potential avenue for achieving elevated radiopharmaceutical concentrations in tumors, a consequence of the protracted exposure and uninterrupted administration of the drug. The continuous infusion approach ensures a sustained and gradual release of the radiopharmaceuticals over time, potentially leading to augmented drug accumulation in the extracellular domain^49^. In contrast, the concentrations of bound and intracellular radiopharmaceuticals under bolus injections are comparatively less pronounced than those attained through continuous infusion. The observed difference in the behavior of radiopharmaceutical distribution patterns between bolus and continuous infusion can be attributed to the kinetics of radiopharmaceutical delivery, clearance, and saturation of PSMA receptors. This difference in concentration dynamics has implications for the therapeutic outcome, as higher radiopharmaceutical concentrations may result in increased efficacy in terms of tumor cell killing and overall treatment response. The current study supports earlier research contrasting bolus injections vs. continuous infusions^50^. created a mathematical model to compare DOX concentrations after bolus injections vs. continuous infusions. In interacellular medicines, similar concentration fluctuations are seen. However, the behavioral pattern of variations in the concentration of exteracellular medication in the tumor differs between the current research and the above-mentioned study^50^. This variance can be attributed to the distinct mechanisms employed by the two drugs for transiting from the extracellular to the intracellular space.

This study has specific limitations and assumptions. First, the current model does not include the geometry of microvessels and heterogeneous distribution. The density of microvessels can directly affect radiopharmaceutical concentration in tumors, which will be covered in our future studies. Second, the model needs to be tested for a larger patient population to prove its robustness. Several biological phenomena are not considered in this study, such as tumor growth and angiogenesis. Physiological parameters and geometry are time-independent since the simulation duration is much shorter than the time scale of tumor growth. Future studies should examine the organs at risk, such as the liver and kidney, to optimize the amount of injected dose. The present model can be personalized by including patient-specific parameters, such as the extravasation rate of ^177^Lu-PSMA-617 from blood microvessels to the interstitial space and porosity of the interstitial space, by using dynamic imaging modalities^51^. This will make model predictions more reliable for personalized therapies, and the model can be used to tailor patient-specific treatment plans.

## Methods

**Fig. 7:**
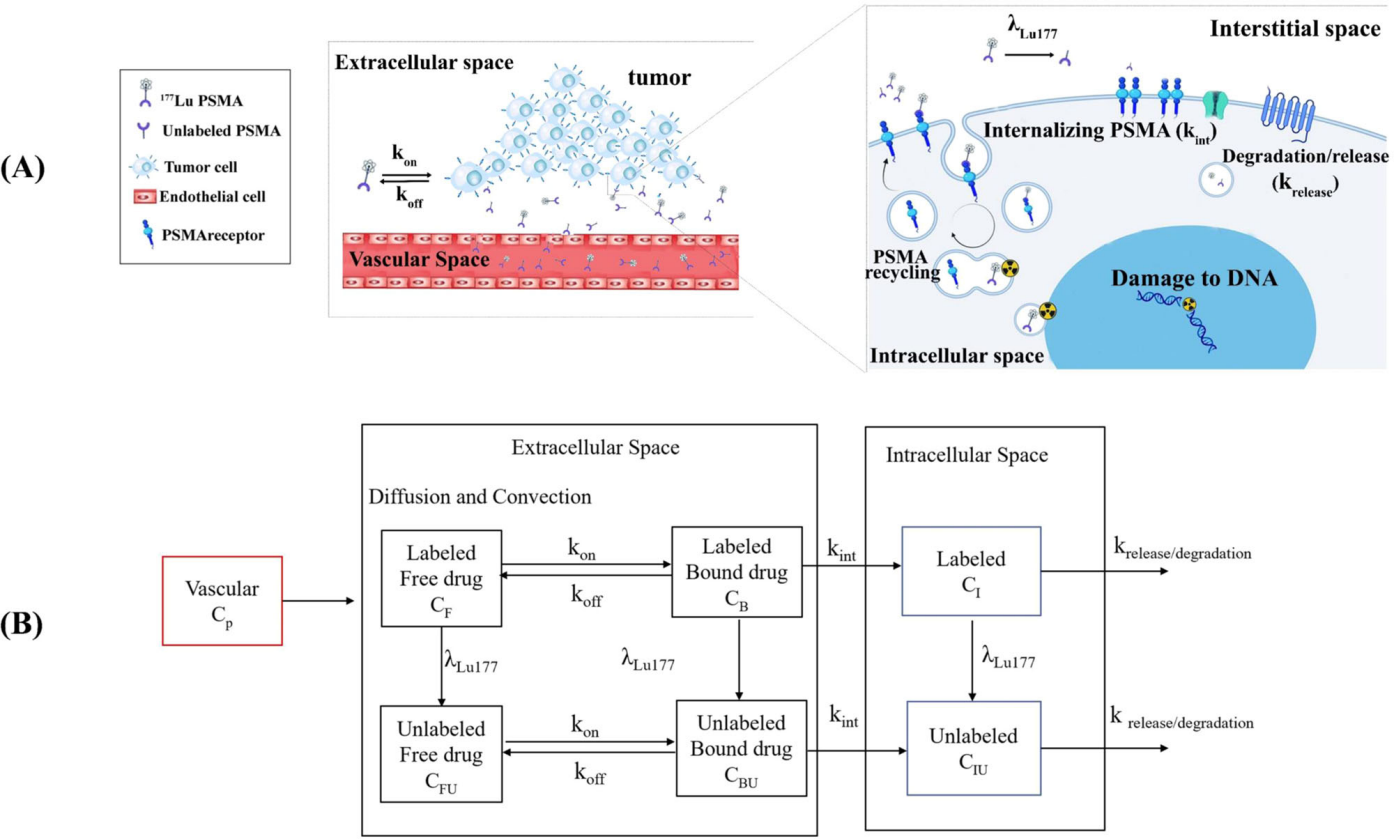
Schematic of radiopharmaceutical transport model. **A** PSMA ligands can associate/disassociate with receptors via k_on_ and k_off_ constant rates. Then, each PSMA is transported to the intracellular compartment by an endocytosed process (k_int)_. The last step is radiopharmaceutical degradation with a k_release/degradation_ rate. Throughout the process, ^177^Lu emits beta (β) particles that can cause damage to DNA. **B** Radiopharmaceutical transport compartment model.

Three-dimensional computational models can be used in various ways to investigate interactions between multiple radiopharmaceutical delivery steps, tumor characteristics, and radiopharmaceutical properties. As seen in Fig. 7A, B, first, the radiopharmaceutical is administered into the blood circulation and reaches the extracellular space via the microvasculature. Next, it is extravasated into the extracellular matrix by convection and diffusion processes. Subsequently, it is transported in extracellular space as a result of diffusion and convection in tissue^52^. The binding of ^177^Lu-PSMA-617 to cell receptors promotes the movement of radiopharmaceutical into its intended location. Afterward, radiopharmaceutical internalization takes place as the next step. The developed model includes all major mechanisms, including the association and dissociation of ligands, internalization of drugs, and degradation of radiopharmaceuticals^53^.

The subsequent sections provide detailed mathematical modeling, including equations governing interstitial fluid flow, drug concentration, radiopharmaceutical transport, the internalization process of PSMA receptors, geometry, boundary conditions, numerical approach, simulations, parameters, and validation of the model.

### Geometry

**Fig. 8:**
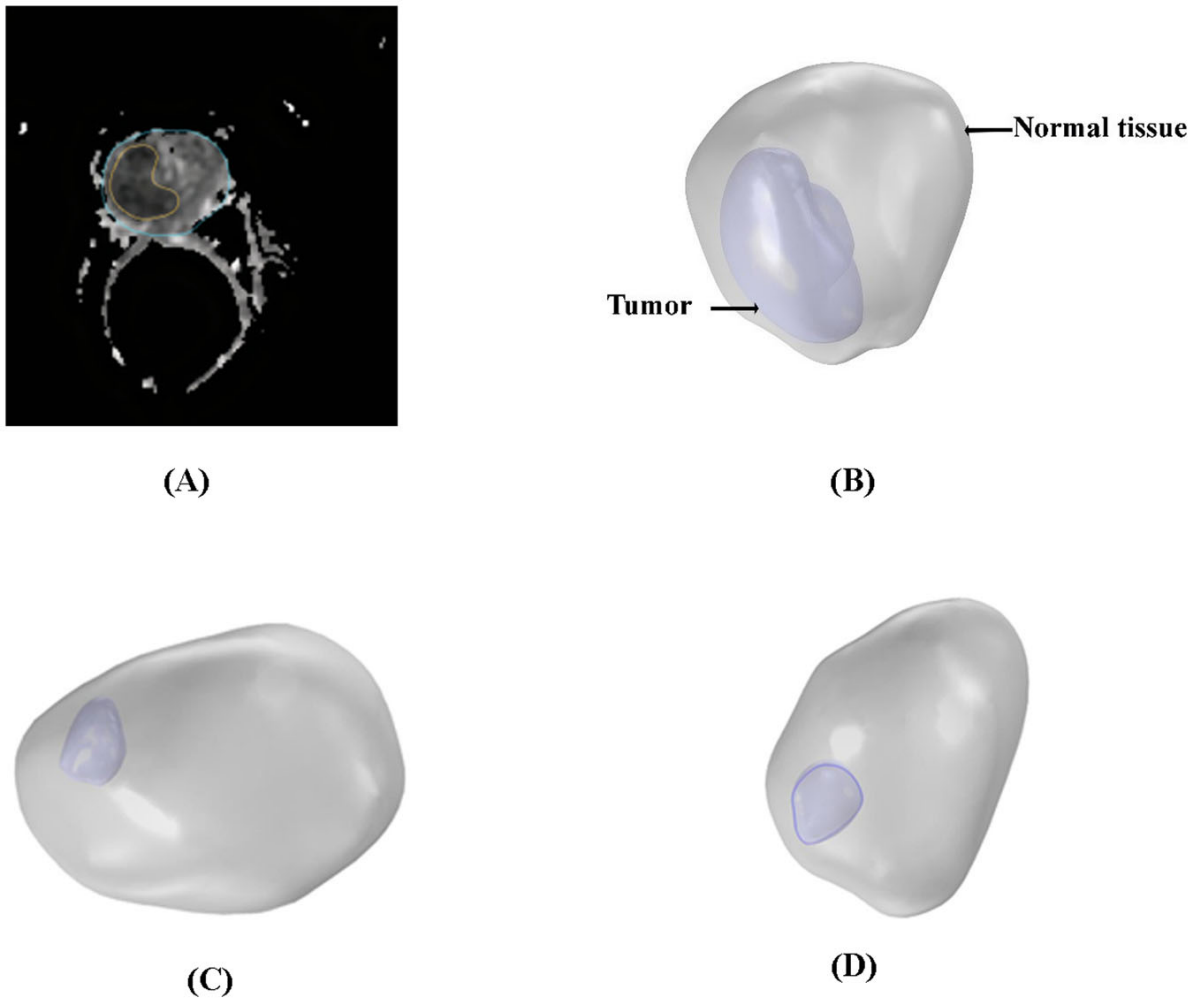
Tumor model reconstruction and grid generation. **A** MRI images from patients with prostate cancer were used to reconstruct tumor models. **B** Three-dimensional reconstruction of tumor 1 (light blue) and normal tissue (light grey). **C** Three-dimensional reconstruction of tumor 2 (light blue) and normal tissue (light grey). **D** Three-dimensional reconstruction of tumor 3 (light blue) and normal tissue (light grey).

**Fig. 9:**
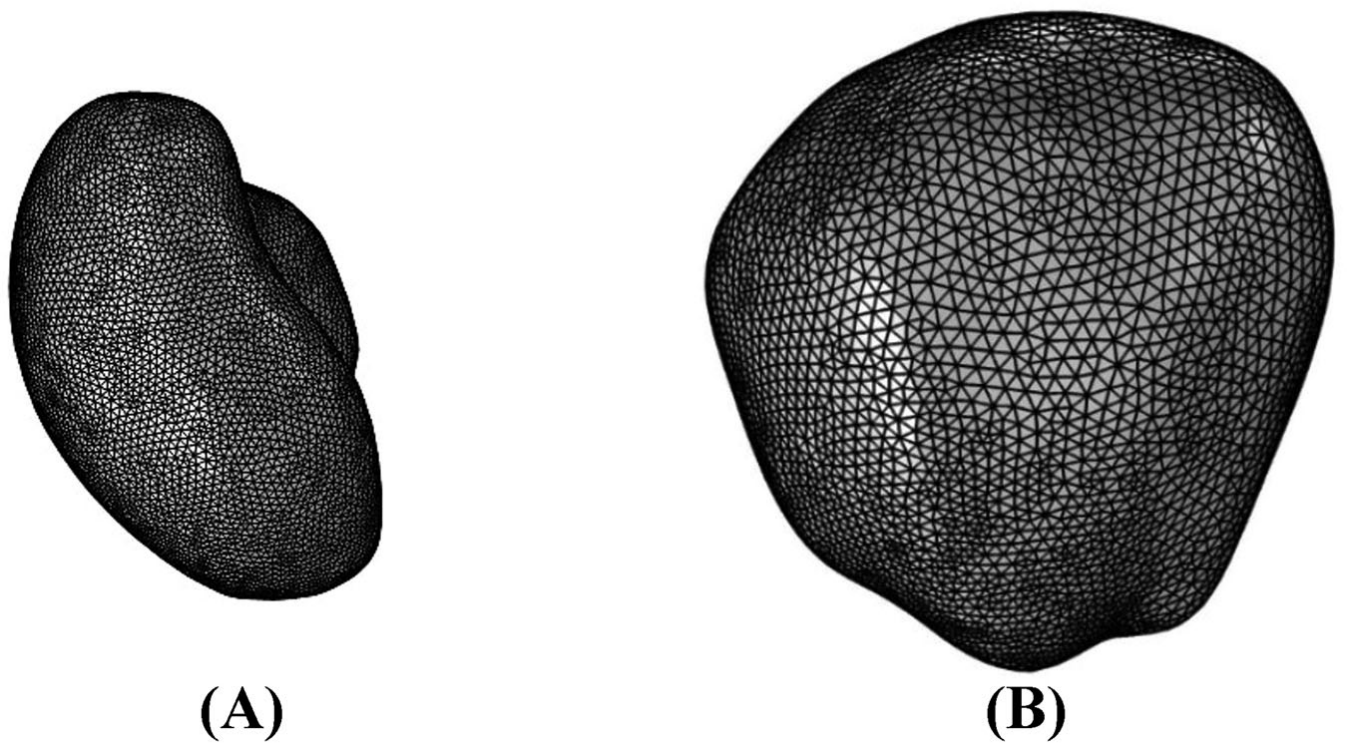
Tumor 1 model grid generation. **A** Grid generation in tumor 1. **B** Grid generation in normal tissue. There are 498,623 tetrahedral elements in the model.

To investigate the distribution of radioligands in tumors using 3-dimensional MRI images for the first time, the 3-dimensional geometry of a human prostate tumor and its surrounding tissue was reconstructed from MR images available in the datasets of the cancer imaging archive (TCIA)^54,55^. Multi-slice anatomical images of the prostate were acquired in 3 orthogonal planes with an echo-planar (EP) sequence, with each image comprising 256 by 256 pixels. Additionally, the field of view, slice thickness, number of sides, and pixel size were all 220.01 mm, 4.00 mm, 32 cm, and 0.859 mm, respectively. Figure 8A shows the MRI image of tumor 1 used for the geometric reconstruction of tumor models. Based on the signal intensity values of the transverse images, the tumor was segmented from its surrounding normal tissue using Materialise Mimics software (Materialise HQ, Leuven, Belgium). Figure 8B–D displays the ultimate geometry of the tumors investigated in this study. Finally, COMSOL Multiphysics version 5.6a (COMSOL Inc., Stockholm, Sweden) was used to generate the computational mesh, as shown in Fig. 9A, B (tumor 1).

The grid independency test is also applied, and the outputs of IFP and ^177^Lu-PSMA concentration for coarse, fine, finer, and extremely fine computational grids are assessed. Finer and coarse grids differ by up to 3%, and finer and extremely fine meshes by about 1%. The finer mesh is considered, and the extra fine mesh is considered for internal boundaries. As a result, concentration gradients can be captured more accurately in tumor 1. Finally, the model consists of 498,623 tetrahedral elements (tumor 1).

**Fig. 10:**
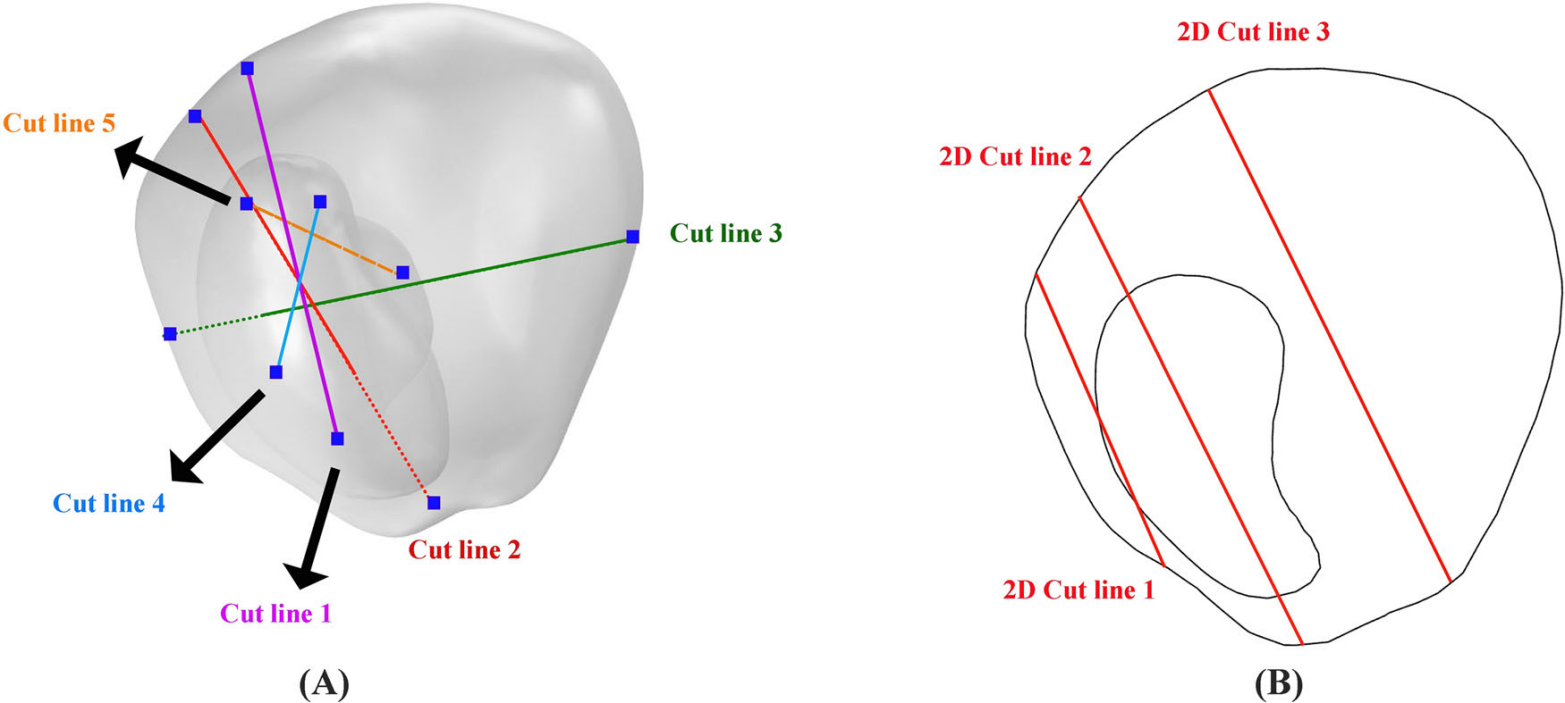
Cutting planes in tumor 1 computational domain. **A** Placement of five distinct cutting planes within the computational domain of tumor 1. These planes are positioned perpendicular to the lines as shown in this fig, selected to improve visualization. **B** The location of 3 cut lines in XY plane in the computational domain of tumor1.

Figure 10A illustrates the positioning of five distinct cutting planes within Tumor 1’s computational domain. These planes are orthogonal to the lines indicated in the Fig. 10A, chosen for enhanced clarity in presentation. Strategically placed, these planes (Fig. 10A) and lines (Fig. 10B) enable the examination of spatial and temporal alterations in radiopharmaceutical concentration, providing valuable insights into the evolving distribution patterns within the tumor.

### Boundary conditions

Boundary conditions are examined by considering two boundaries: between the cancerous tissue and the normal tissue, called the internal border, and the boundary outside the healthy tissue, known as the external border. Summarized in Table 1 are the boundary conditions that are applied to the interstitial fluid flow equations and the solute transport equations^56^. At the interface between tumor and normal tissue, flow and flux continuity have been imposed. In addition, at the outer boundary, the IFP has been assumed to be zero as far away from the tumor boundary in the normal tissue the IFP is zero. Further zero outflow boundary condition has been imposed on the outer boundary of the normal tissue, assuming that the drug does not cross the outer boundaries of the tissue. n is the normal vector. Initially, the drug concentration has been assumed to be zero in the tissue.

**Table 1:** The boundary conditions of the present study for fluid flow and concentration distribution

Region	Fluid flow	Concentration
Boundary between tumor and normal tissues	${\left.-{K}^{t}\nabla {P}_{i}\right|}_{{\varOmega }^{t}}={\left.-{K}^{n}\nabla {P}_{i}\right|}_{{\varOmega }^{n}}$	${\left.{-D}_{{eff}}^{t}\nabla C+{v}_{i}C\right|}_{{\varOmega }^{t}}={\left.{-D}_{{eff}}^{n}\nabla C+{v}_{i}C\right|}_{{\varOmega }^{n}}$
	${\left.{P}_{i}\right|}_{{\varOmega }^{t}}={\left.{P}_{i}\right|}_{{\varOmega }^{n}}$	${\left.C\right|}_{{\varOmega }^{t}}={\left.C\right|}_{{\varOmega }^{n}}$
Outer boundary	${P}_{i}$ = 0	$-n\,\cdot\, \nabla {\rm{C}}=0$

• Ω^t^ and Ω^n^ demonstrates the tumor and normal tissue at the boundary, respectively.

• n is the normal vector.

### Interstitial fluid flow

Solving interstitial fluid flow models establishes a biomechanical environment for drug delivery. Darcy’s law describes the fluid flow in tumor and normal tissues well, one of the first formulas to describe the fluid flow in porous media. It has been assumed that tumors are porous because inter-capillary distances are orders of magnitude less than scales of drug transport in tissues. Different biological tissues can benefit from applying this equation to the relation between IFP and interstitial fluid velocity (IFV). Assuming that tissue is porous, IFV is superficial IFV averaged over the whole representative elementary volume. In a tissue, then, the fluid equation is given below^57^:

1 $${\boldsymbol{v}}=-\frac{k}{\mu }\nabla {p}_{i}$$

The IFV and IFP are represented by v and pi, respectively. The mass conservation equation for interstitial fluid is given by assuming interstitial fluid to be incompressible and Newtonian^57^:

2 $$\nabla\, \cdot\, {\boldsymbol{v}}={\varphi }_{B}-{\varphi }_{L}$$

In Eq. 2, ${\varphi }_{B}$ represents the source terms accounting for the influx of interstitial fluid from blood capillaries, and ${\varphi }_{L}$ is the sink term representing the rate at which the lymphatics absorb fluid. According to Starling’s law, ${\varphi }_{B}$ and ${\varphi }_{L}$ may be calculated:

3 $${\varphi }_{B}={K}_{B}\frac{S}{V}\left[{p}_{B}-{p}_{i}-{\sigma }_{T}({\pi }_{B}-{\pi }_{i})\right]$$

4 $${\varphi }_{L}={K}_{L}\frac{{S}_{L}}{V}\left[{p}_{i}-{p}_{L}\right]$$

where ${K}_{B}$ represents the hydraulic conductivity of the microvessel wall, $S/V$ denotes the surface area of blood vessels per unit volume of tissue, ${p}_{B}$ is the vascular pressure, ${\sigma }_{T}$ represents the average osmotic reflection coefficient, ${\pi }_{B}$ is the osmotic pressure of plasma, and ${\pi }_{i}$ is that of the interstitial fluid.

There is no consideration of the lymphatic system when dealing with tumor tissue^21^. According to Eq. 4, lymphatic walls’ hydraulic conductivity, the surface area of lymphatic vessels per unit volume of tissue, and the intralymphatic pressure are represented by ${K}_{L}$, ${S}_{L}/V$, and ${p}_{L}$, respectively.

### Time and length scales

The equations for the concentration of the free drugs in the interstitial space of the tissue are as follows^58^:

5 $${C}_{F}\left(x\right)=-{A}_{F}\,\exp \left(-\frac{x}{{L}_{F}}\right)$$

where the characteristic length scale is given by ${L}_{F}=\frac{2D}{{({v}^{2}+4{K}_{F}D)}^{1/2}-v}$, and ${K}_{F}={D}_{R}{k}_{{on}}$. *kon* is the binding rate constant, *DR is receptor density*, *D* is the diffusion coefficient, and *v* is the interstitial fluid velocity of the interstitial space. In solid tumors’ interiors, the uniformly elevated fluid pressure often eliminates interstitial flow; thus, the characteristic length becomes ${L}_{F}={\left(\frac{D}{{K}_{F}}\right)}^{1/2}$.

Furthermore, the characteristic diffusion time scale (T_d_) and the characteristic advection time scale (T_a_) are described as follows:

6 $${{\rm{T}}}_{{\rm{d}}}=\frac{{{L}_{F}}^{2}}{D}$$

7 $${{\rm{T}}}_{{\rm{a}}}=\frac{{T}_{d}}{{Pe}}$$

### ^177^Lu-PSMA concentration

#### Plasma pharmacokinetics

Depending on the infusion method, the equations take different forms. According to the plasma pharmacokinetics of ^177^Lu-PSMA, the radiopharmaceutical concentration following bolus injection decays exponentially^37,59^.

8 $${Cp}=\frac{A}{V}{e}^{-\alpha t}$$

where $A$ is the initial injection amount of ^177^Lu-PSMA, $V$ is the compartmental parameter, $\alpha$ is the compartmental clearance rate, and $t$ is the time.

The following equation can be used to model continuous infusion^59^:

9 $${Cp}\left(t\right)=\frac{A}{V\alpha T}\left(1-{e}^{-\alpha t}\right)t\, <\, T$$

10 $${Cp}\left(t\right)=\frac{A}{V\alpha T}\left({e}^{\alpha T}-1\right){e}^{-\alpha t}t\ge T$$

where *T* is the infusion duration. Furthermore, the alpha value ($\alpha$) was determined by fitting the plasma concentration-time profile of ^177^Lu-PSMA.

#### Radiopharmaceutical transport

In the interstitial fluid, free and bound radiopharmaceuticals are transported according to CDR equations. A description of the free drug, bound drug, and the intracellular concentration of the hot and cold parts are as follows^36,60^:

Radiolabeled (hot):

11 $$\frac{\partial {C}_{F}}{\partial t}=-\,\nabla \cdot\, ({\boldsymbol{v}}{R}_{f}{C}_{F})+\,\nabla\, \cdot (D\nabla {C}_{F})-{\lambda }_{{lu}177}{C}_{F}-(F{{\_}}s){k}_{{on}}{C}_{F}+{k}_{{off}}{C}_{B}+({\Phi }_{B}-{\Phi }_{L})$$

12 $$\frac{\partial {C}_{B}}{\partial t}=-{\lambda }_{{lu}177}{C}_{B}+({F}{{\_}}{s}){k}_{{on}}{C}_{F}-{k}_{{off}}{C}_{B}-{k}_{\mathrm{int}}{C}_{B}$$

13 $$\frac{\partial {C}_{I}}{\partial t}=-{\lambda }_{{lu}177}{C}_{I}-{k}_{{release}}{C}_{I}+\frac{{{FV}}_{i}}{{{FV}}_{c}}{k}_{\mathrm{int}}{C}_{B}$$

Not radiolabeled (cold):

14 $$\frac{\partial {C}_{{FU}}}{\partial t}=-\nabla\, \cdot\, ({\boldsymbol{v}}{R}_{f}{C}_{{FU}})+\nabla\, \cdot\, \left(D\nabla {C}_{{FU}}\right)+{\lambda }_{{lu}177}{C}_{F}-\left({F}{\_}{s}\right){k}_{{on}}{C}_{{FU}}+{k}_{{off}}{C}_{{BU}}+({\Phi }_{{BU}}-{\Phi }_{{LU}})$$

15 $$\frac{\partial {C}_{{BU}}}{\partial t}=+{\lambda }_{{lu}177}{C}_{B}+\left({F}{\_}{s}\right){k}_{{on}}{C}_{{FU}}-{k}_{{off}}{C}_{{BU}}-{k}_{\mathrm{int}}{C}_{{BU}}$$

16 $$\frac{\partial {C}_{{IU}}}{\partial t}=+{\lambda }_{{lu}177}{C}_{I}-{k}_{{release}}{C}_{{IU}}+\frac{{{FV}}_{i}}{{{FV}}_{c}}{k}_{\mathrm{int}}{C}_{{BU}}$$

where ${C}_{F}$ and ${C}_{{FU}}$ are free-labeled and unlabeled PSMA-targeted ligands, respectively. *C*_*B*_ and *C*_*BU*_ represent bound labeled and unlabeled ligands. ${C}_{I}$ and ${C}_{{IU}}$ are internalized labeled and unlabeled ligands, respectively. *D* is the diffusion coefficient of the free radiopharmaceutical, *v* denotes the interstitial fluid velocity field, and *λ*_*lu177*_ is the physical decay. *K*_*on*_ and *K*_*off*_ are the association/dissociation rates, *k*_*int*_ is the internalization rate, and *F*_*s* is equal to free receptors on the cell surface. *K*_*release*_ is the release rate, and *FV*_*i*_ and *FV*_*c*_, respectively, represent the fractional volumes of tumor interstitium and cells within a voxel. Finally, ϕ_*B*,_ ϕ_*BU*_, ϕ_*L*_ and ϕ_*LU*_ represent the solute transport rates of labeled and unlabeled PSMAs from capillaries and lymphatic vessels, which are respectively calculated using the following equations^22,37^:

- Sink term (lymphatic vessels)

17 $${{\rm{\phi }}}_{L}={\varphi }_{L}{C}_{F}$$

18 $${{\rm{\phi }}}_{{LU}}={\varphi }_{L}{C}_{{FU}}$$

- Source term (capillaries)

19 $${{\rm{\phi }}}_{B}={\varphi }_{B}{(1-{\sigma }_{f})C}_{p}+\frac{{PS}}{V}({C}_{P}{-C}_{F})\frac{{Pe}}{{e}^{{Pe}}-1}$$

20 $${{{\phi }}}_{{BU}}={\varphi }_{B}{(1-{\sigma }_{f})C}_{p}+\frac{{PS}}{V}({C}_{P}{-C}_{{FU}})\frac{{Pe}}{{e}^{{Pe}}-1}$$

σ_f_ is the coefficient of filtration reflection, and *P* is the capillary permeability. Pe is the trans-capillary Peclet number defined as follows^21^:

21 $${Pe}=\frac{{\varphi }_{B}(1-{\sigma }_{f})V}{{PS}}$$

### Modeling the internalization process of PSMA receptors

**Fig. 11:**
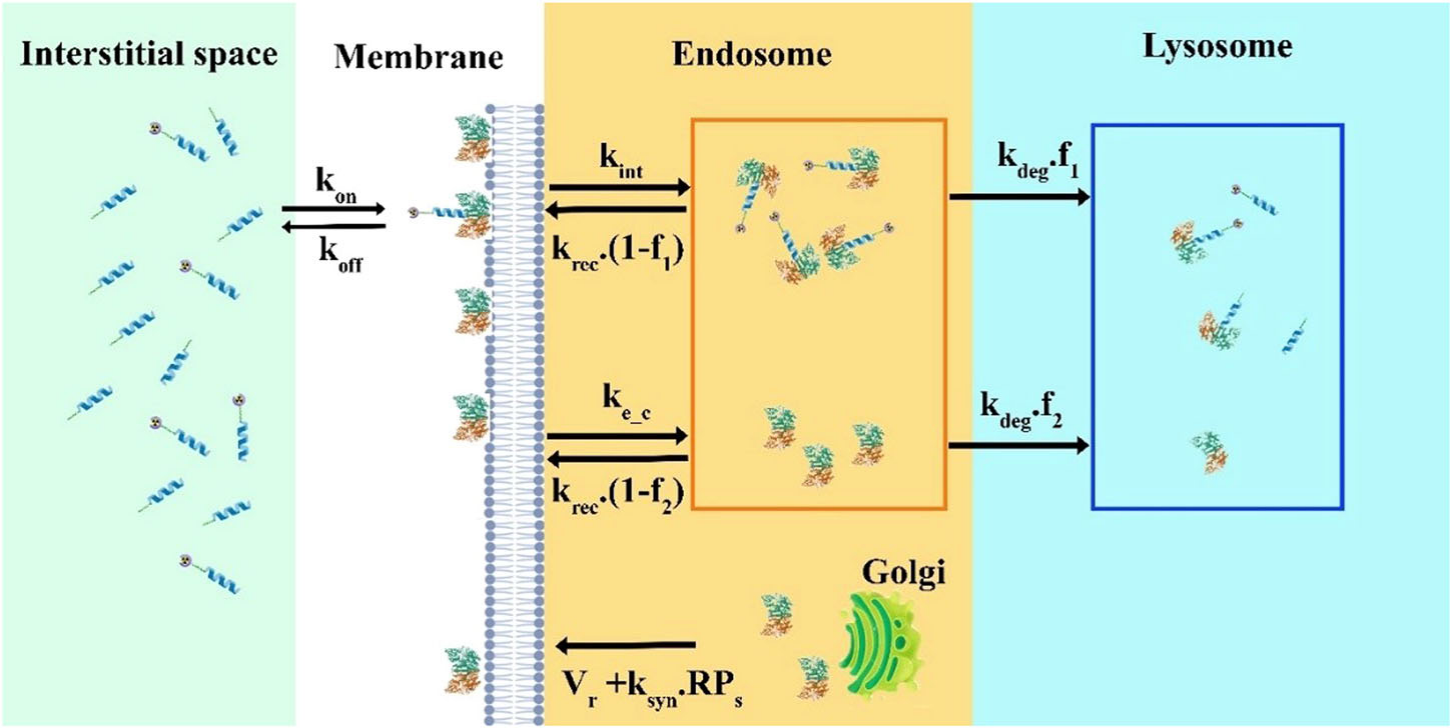
Model structure. PSMA ligands in interstitial space associate/disassociate reversibly to free PSMA receptors with the rate of k_off_ and k_on_. Ligand receptors and free PSMAs are endocytosed with the rate constant of k_int_ and k_e,c_, respectively. During internalization, a fraction of PSMA is recycled back to the plasma membrane at a constant rate (k_rec_). The remaining receptors (f_1_, f_2_) are degraded in the maturing endosome and eventually in the lysosome (k_deg_). Total PSMA receptor biosynthesis combines constitutive V_r_ and ligand-induced synthesis k_syn_.

The extracellular PSMA-targeted ligand binds reversibly to free surface receptors (k_on_/k_off_). Ligand receptors and free receptors can be endocytosed, though the former are internalized more slowly. The sorting endosome brings together internalized free and ligand-bound receptors^61^. In the sorting endosome, a fraction of the internalized PSMA receptors is pinched off with tubular buds and returned to the plasma membrane at a constant k_rec_. Eventually, lysosomes degrade the remaining internalized receptors (k_deg_). The Golgi apparatus continually synthesizes new receptors and sends them to the surface at the rate of V_r_ (Fig. 11). It only considers the rate-restricting processes of the growth factor-induced endocytosis and ignores rapid mechanisms, such as dimerization of the surface receptors, activation of the occupied receptors, and binding to the surface proteins^44^. When this compartmental structure is interpreted according to the law of mass conservation, the following differential equations result^44,62^:

Free surface receptors:

22 $$\frac{\partial F{{\_}}s}{\partial t}=-{k}_{{on}}\left({F}{\_}{s}\right)\left({C}_{{FU}}+{C}_{F}\right)+{V}_{r}+\left({k}_{{syn}}+{k}_{{off}}\right){{RP}}_{s}-{k}_{e,c}.{F}{\_}{s}+(1-{f}_{2}){k}_{{rec}}{F}{\_}{e}$$

Free endosome receptors:

23 $$\frac{{\partial F}{\_}{e}}{\partial t}={k}_{e,c}\left({F}{\_}{s}\right)-\left(\left(1-{f}_{2}\right){k}_{{rec}}+{f}_{2}{k}_{{deg}}\right){F}{\_}{e}$$

Occupied surface receptors:

24 $$\frac{{\partial {RP}}_{s}}{\partial t}={k}_{{on}}\left({F}{\_}{s}\right)\left({C}_{{FU}}+{C}_{F}\right)+\left(1-{f}_{1}\right){k}_{{rec}}{{RP}}_{e}-({k}_{{off}}+{k}_{\mathrm{int}}){{RP}}_{s}$$

Occupied endosome receptors:

25 $$\frac{{\partial {RP}}_{e}}{\partial t}=-\left(\left(1-{f}_{1}\right){k}_{{rec}}+{f}_{1}{k}_{{deg}}\right){{RP}}_{e}+{k}_{\mathrm{int}}{{RP}}_{s}$$

### Numerical approach, and simulations

To solve this problem, there are two different steps: steady-state and time-dependent. In the steady-state step, Darcy’s law is solved to obtain the IFP and IFV. Following that, equations for solute transport that are time-dependent are solved. Finite element analysis is utilized to assess the coupled nonlinear set of governing equations as well as the boundary conditions. In this study, COMSOL Multiphysics 5.6a (COMSOL, Inc., Burlington, MA, USA) was used as the simulation software. The equations are solved using a segregated approach with a time-step of 0.1 min and a relative tolerance of 0.001. This time step has been taken after a time sensitivity test. For temporal derivatives, the BDF method was used, and initialization of the solution was done using the Backward Euler method. Regarding the discretization of the concentration, a linear method was chosen since it provided a sufficient convergence error for the software to converge. Drug delivery time (physical time) is considered 50 hours for analysis. Furthermore, the MUMPS solver was used to solve equations. An Intel Core i9-11900H @ 2.50 GHz with 32 GB RAM was employed for the numerical simulations.

A detailed discussion of each factor’s effects is conducted following the verification of the model. The following factors are examined:

- The increase in injection amount from 100 to 1000 [nmol]
- Changes in density receptors between 10 to 500 [nmol.L^−1^]
- The decrease in the receptors’ recycling rate from 10^−1^ to 10^−4^ [min^−1^]
- The continuous infusion vs bolus
- Increase labeled peptides amount from 1 to 10% of total injection amount.

A further and more detailed investigation will be conducted on the most influential parameters on the concentration of ^177^Lu-PSMA. The concentrations of radiopharmaceutical are non-dimensionalized by C_P0_ (initial concentration of drug in plasma). Moreover, characteristic timescales and length scales are examined.

### Parameters of model

The model parameters are of paramount significance in elucidating the behavior of drugs within both healthy and tumor tissues. Within Table 2, a comprehensive depiction of the drug’s distinct characteristics is meticulously provided, unveiling crucial properties that dictate its intricate interactions within the biological milieu. Moreover, Table 3 offers a thorough compilation of the requisite values to decode the complexities of interstitial fluid flow.

**Table 2:** Values of the parameters used in the model. (Ref. ^10,21,36,62,68^)

Parameter	Value	Ref.	Definition
D_eff_	8.7 × 10^−7^ [cm2 s^−1^]	^36^	Coefficient of effective diffusion
Tumor 1-Density_receptor_	50 [nmol·l^–1^]	^10^	Receptors density
Tumor 2-Density_receptor_	500 [nmol·l^–1^]	^10^	Receptors density
Tumor3- Density_receptor_	200 [nmol·l^–1^]	^10^	Receptors density
Normal cellDensity_receptor_	0.1* Tumor_Density_receptor_	^10^	Receptors density of normal cells
f1	0.5	^62^	Fraction of occupied receptors sorted to degradation
f2	0.5	^62^	Fraction of free receptors sorted to degradation
FVc	61%	^36^	Fractional cellular volume
FVi	39%	^36^	Fractional interstitial volume
α	0.0521 [1/h]	fit	Compartmental clearance rate
λ_lu177_	7.15 × 10^–5^ [Min^–1^]	^10^	Physical decay ^177^Lu
K_int_	0.001[Min^–1^]	^10^	Internalization rate of the cell through the receptors
k_on_	0.046 [L/nmol/min]	^10^	Drug binding rate to cell receptors
k_off_	0.046[Min^–1^]	^10^	Drug unbinding rate from cell receptors
k_rel_	2 × 10^–4^ [Min^–1^]	^10^	Release rate
k_deg_	2.9891 × 10^–4^ [1/s]	^62^	Degradation rate of receptors
k_e,c_	0.007 [min^−1^]	^62^	Constitutive endocytosis
k_rec_	0.15[min^−1^]	^62^	Recycling rate of receptors
k_syn_	0.0118 [min^−1^]	^62^	Ligand induced synthesis
P	3.3 × 10^−4^ [cm s^−1^]	^36^	Vessel wall permeability
R_f_	1	^36^	Molecule/Carrier movement coefficient
Vr	1.2 × 10^−22^ [mol/(m^3^s)]	^62^	Constitutive synthesis
σ_f_	0.9	^21,68^	Coefficient of filtration reflection

**Table 3:** Values of the parameters used in the modeling of interstitial fluid flow. (Ref. ^28,69^)

Parameter	Tissue	Value	Ref.
K [cm2/mmHgs]	Normal	8.53 × 10^−9^	^69^
	Tumor	4.13 × 10^−8^	^69^
Lp [cm/mmHgs]	Normal	0.36 × 10^−7^	^28,69^
	Tumor	2.80 × 10^−7^	^28,69^
L_PL_ S_L_/V [1/mmHg s]	Normal	1.33 × 10^−5^	^28^
P_B_[mmHg]	Both	15.6	^69^
P_l_	normal	0	^28^
S/V[cm^−1^]	Normal	70	^69^
	Tumor	200	^69^
Π_B_[mmHg]	Both	20	^69^
Π_i_[mmHg]	Normal	10	^69^
	Tumor	15	^69^
${\sigma }_{s}$	Normal	0.91	^69^
	Tumor	0.82	

### Validation of the model

**Fig. 12:**
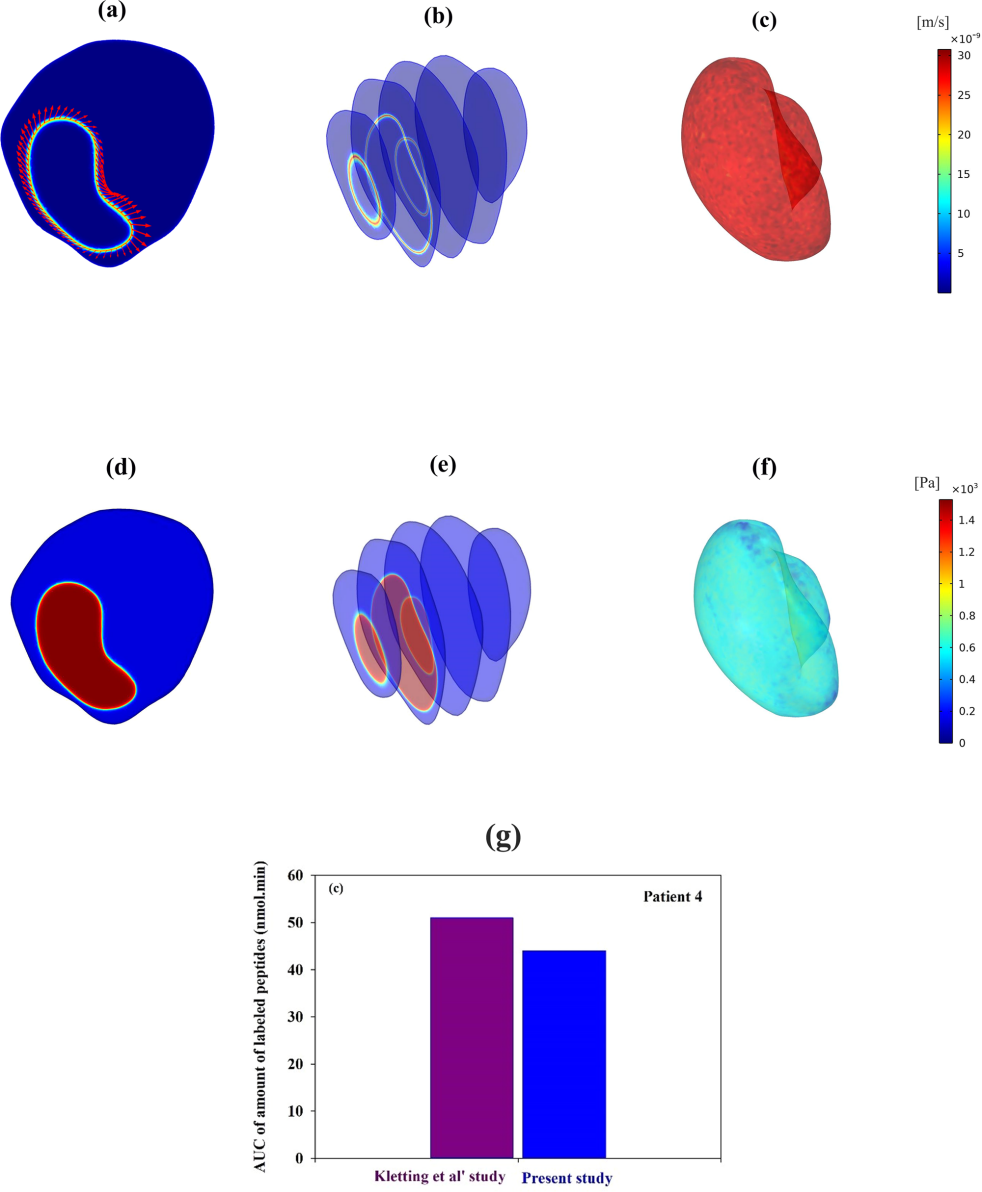
Validation of simulation results. **a** A spatial interstitial fluid velocity in tumor and normal tissue (IFV). The red arrows at the tumor’s perimeter indicate that interstitial fluid is directed toward normal tissue from the tumor’s periphery. **b** IFV in five cross-sections of tumor 1 and normal tissue. **c** Three-dimensional surface visualization of IFV in tumor 1. **d** There is a spatial interstitial fluid pressure (IFP). **e** IFP in five cross-sections of tumor 1 and normal tissue. **f** Three-dimensional surface visualization of IFP in tumor 1. **g** Between the present model and the Kletting model^10^, there is a 13.5% difference in the area under the curve of the amount of labeled PSMA (AUC).

Interstitial fluid pressure (IFP) and interstitial fluid velocity (IFV) significantly influence pharmacological substance distribution in the body via the convection process. The current model predicts IFV similarly compared to other models (Fig. 12a, b)^63–66^. Tumor and normal tissue IFPs are measured as 1490.8 Pa and 40 Pa, consistent with prior mathematical analysis as well as with the experimental evidence (Fig. 12d, e)^21,67^. In an effort to enhance comprehension regarding the distribution and visualization of IFP and IFV, Fig. 12c, f depict their spatial patterns across the surface of Tumor 1. It is worth highlighting that the most substantial IFV variations, as dictated by Darcy’s law, manifest at the interface between the tumor and adjacent normal tissue^22^.

In this area, there has only been a handful of in silico and experimental research carried out. We selected the study by Kletting et al.^10^ as the validation reference for its relevance and complementary nature to our current research. Their work employs a pharmacokinetic modeling approach using a PBPK model to investigate the distribution of ^177^Lu-PSMA in tumors. This aligns with the objective of the present study, which also focuses on drug distribution in solid tumors using a spatiotemporal drug delivery model. The tumor characteristics in both studies were carefully matched. However, the AUC of concentration of the labeled PSMA in our present model was found to be 13.5% lower than that predicted by the PBPK model (Fig. 12g). This difference can be attributed to the fundamental distinction between the two models’ mathematical foundations, with the PBPK model employing ordinary differential equations (ODEs) and our current model utilizing partial differential equations (PDEs). Moreover, our study benefits from the incorporation of real tumor geometry, leading to increased accuracy by accounting for specific tumor characteristics, such as size and location. These variations in methodology and consideration of tumor-specific features could account for the observed differences in the area under the concentration curve when comparing the results of our study and Kletting et al.‘s investigation^10^.

### Reporting summary

Further information on research design is available in the Nature Research Reporting Summary linked to this article.

### Supplementary information

Supplementary Material

reporting summary

## Data Availability

All data used for this study are available from the corresponding author upon request.

## Code availability

All data used for this study are available from the corresponding author upon request.
