# Supplementary material for: Radiopharmaceutical transport in solid tumors via a 3-dimensional image-based spatiotemporal model
Source: NPJ Syst Biol Appl. 2024 Apr 12;10:39. doi: 10.1038/s41540-024-00362-4 (PMC11015041; doi:10.1038/s41540-024-00362-4)
Supplement: Supplementary file 1 — Supplementary Material [file 41540_2024_362_MOESM1_ESM.pdf]

Supplementary File for

Radiopharmaceutical Transport in Solid Tumors *via* a 3-Dimensional Image-Based Spatiotemporal Model

Anahita Piranfar[1], Farshad Moradi Kashkooli[1], Wenbo Zhan[2], Ajay Bhandari[3], Babak Saboury[4,5], Arman Rahmim[5,6], M. Soltani[1,5,7,8*]

[1] Department of Mechanical Engineering, K. N. Toosi University of Technology, Tehran, Iran
[2] School of Engineering, King's College, University of Aberdeen, Aberdeen AB24 3UE, UK
[3] Biofluids Research Lab, Department of Mechanical Engineering, Indian Institute of Technology (Indian School of Mines), Dhanbad 826004, India
[4] Department of Computational Nuclear Oncology, Institute of Nuclear Medicine, Bethesda, USA
[5] Department of Integrative Oncology, BC Cancer Research Institute, Vancouver, BC, Canada
[6] Departments of Radiology and Physics, University of British Columbia, Vancouver, BC, Canada
Department of Electrical and Computer Engineering, University of Waterloo, ON, Canada
[8] Centre for Biotechnology and Bioengineering (CBB), University of Waterloo, Waterloo, ON, Canada
[*] Corresponding author, Email: msoltani@uwaterloo.ca (M. Soltani), Tel./Fax: +1 (519) 888-4567.

This file includes:

Supplementary Figure 1. Spatiotemporal distribution of free (C_F), Bound (C_B), internalized (C_I), and total (C_F+C_B+C_I) concentration of labeled PSMA at different times.

Supplementary Figure 2. Spatiotemporal Distribution of Total (C_F+C_B+C_I) Concentration of Labeled PSMA Along Four Distinct Cut planes in the Computational Domain of Tumor 1 at Different Time Points.

Supplementary Figure 3. Temporal Changes in Concentration for Three XY Plane Cut Lines.

Supplementary Figure 4. Spatial Distribution of Radiopharmaceutical Concentration along Cutline 2 (2D cut line).

Supplementary Figure 5. Investigating the effects of the administered radiopharmaceutical amount on TIA (For tumor2 and 3). Increasing the injected quantity of labeled and unlabeled ligands initially enhances TIA, but at larger amount, receptor saturation caused by unlabeled ligands results in a reduction in TIA.

Supplementary Figure 6. Exploring the impact of the labeled PSMA-617 percentage on TIA in the tumor. The findings illustrate a notable rise in TIA with an increase in the percentage of labeled PSMA from 1% to 10%.

Results

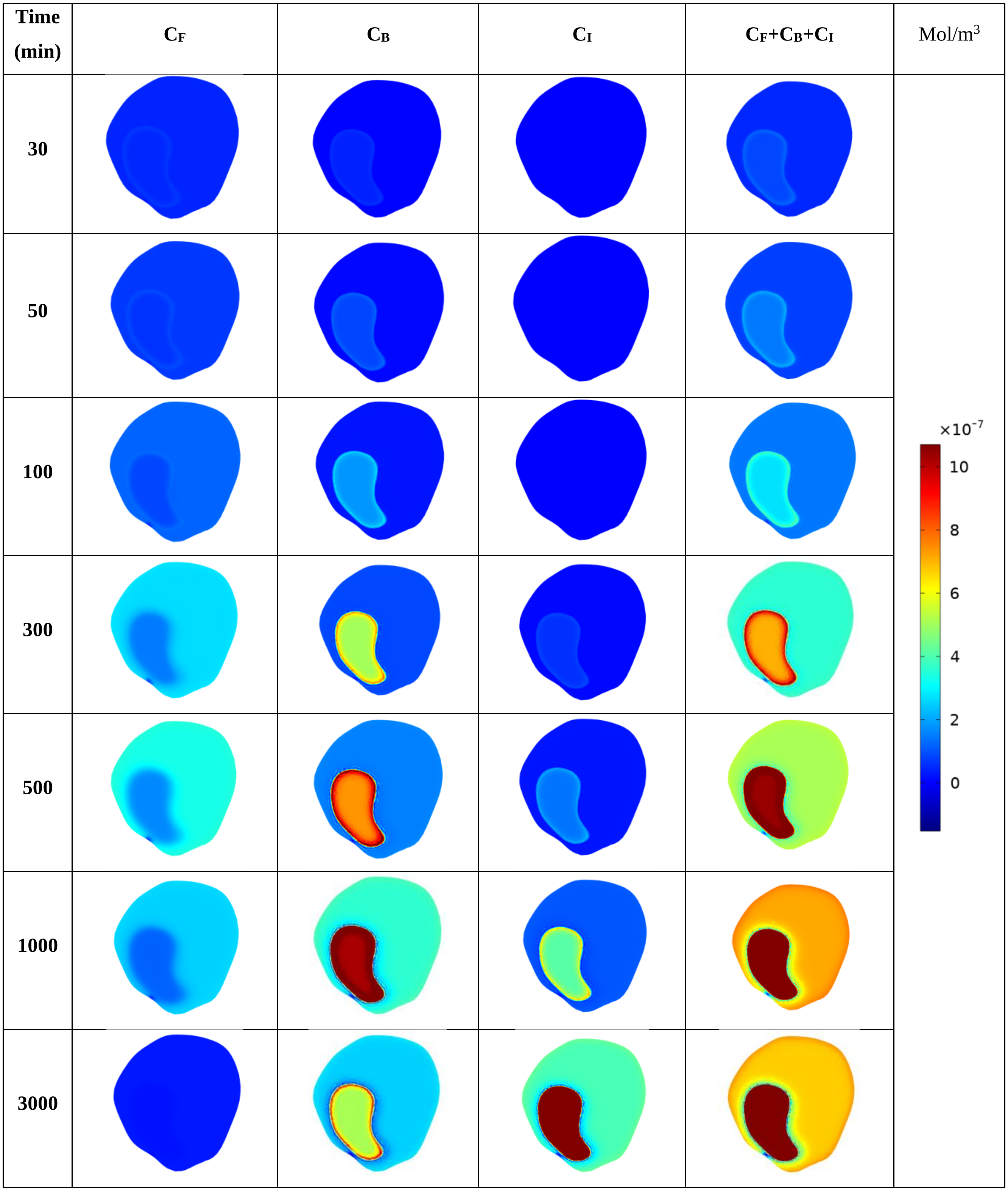
Time (min)
CF
CB
CI
CF+CB+CI
Mol/m3
30
50
100
300
500
1000
3000
×10-7
10
8
6
4
2
0

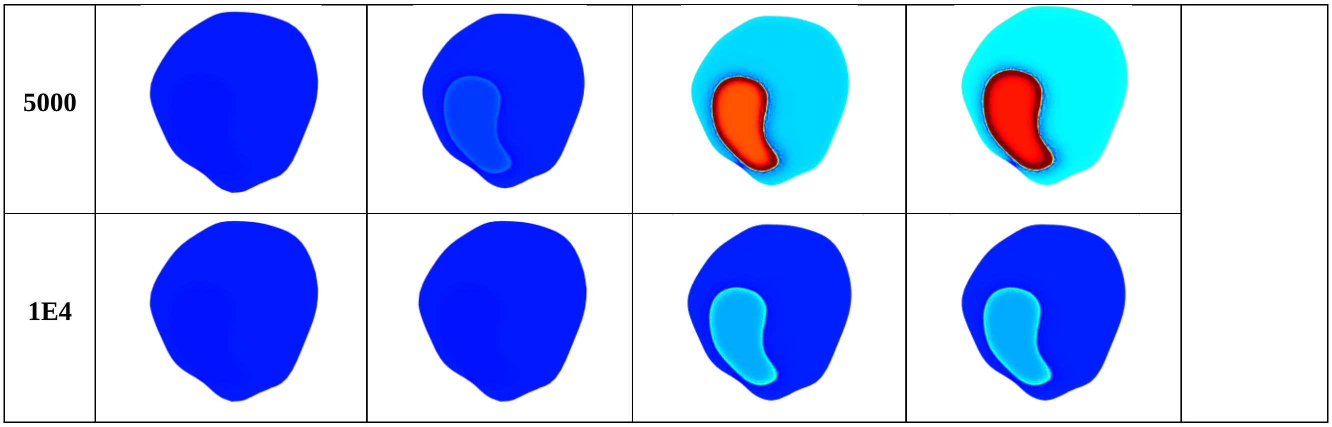

Supplementary Figure 1. Spatiotemporal distribution of free (C_F), Bound (C_B), internalized (C_I), and total (C_F+C_B+C_I) concentration of labeled PSMA at different times.

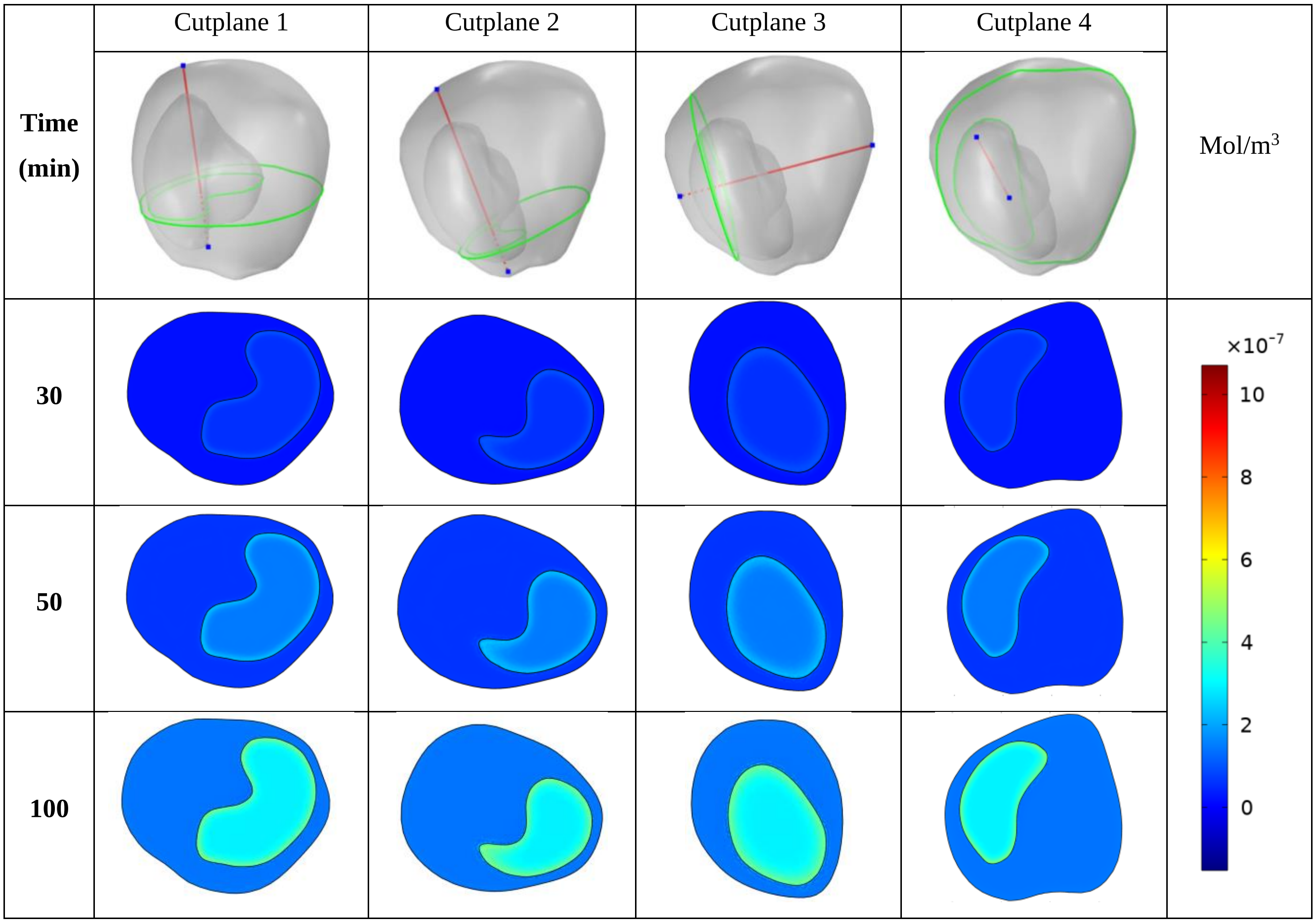

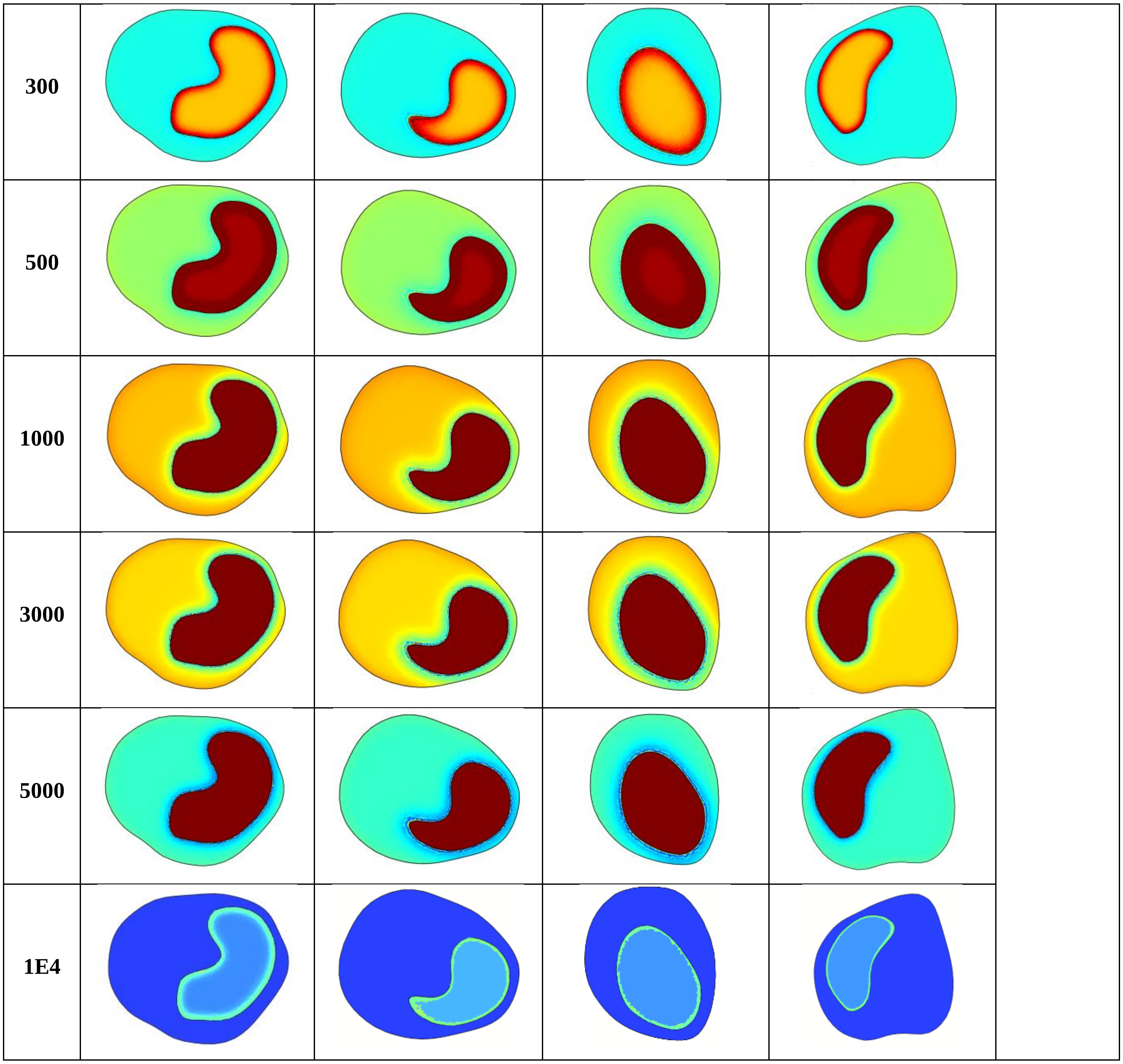

Supplementary Figure 2. Spatiotemporal Distribution of Total (C_F+C_B+C_I) Concentration of Labeled PSMA Along Four Distinct Cut planes in the Computational Domain of Tumor 1 at Different Time Points

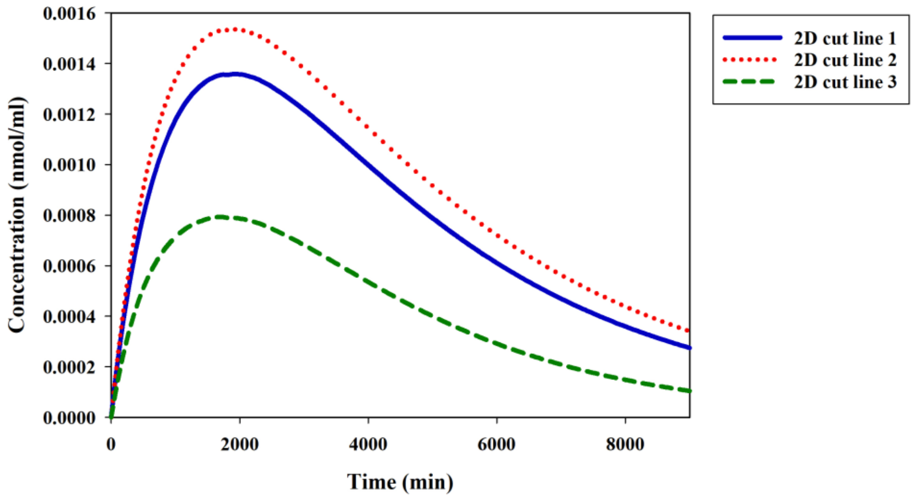

Supplementary Figure 3. Temporal Changes in Concentration for Three XY Plane Cut Lines.

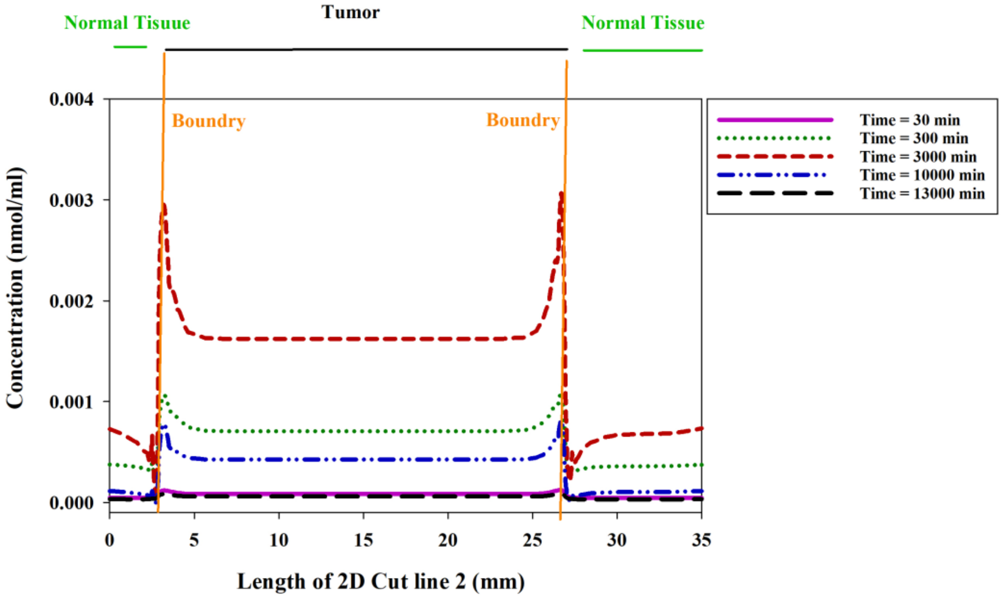

Supplementary Figure 4. Spatial Distribution of Radiopharmaceutical Concentration along Cutline 2 (2D cut line).

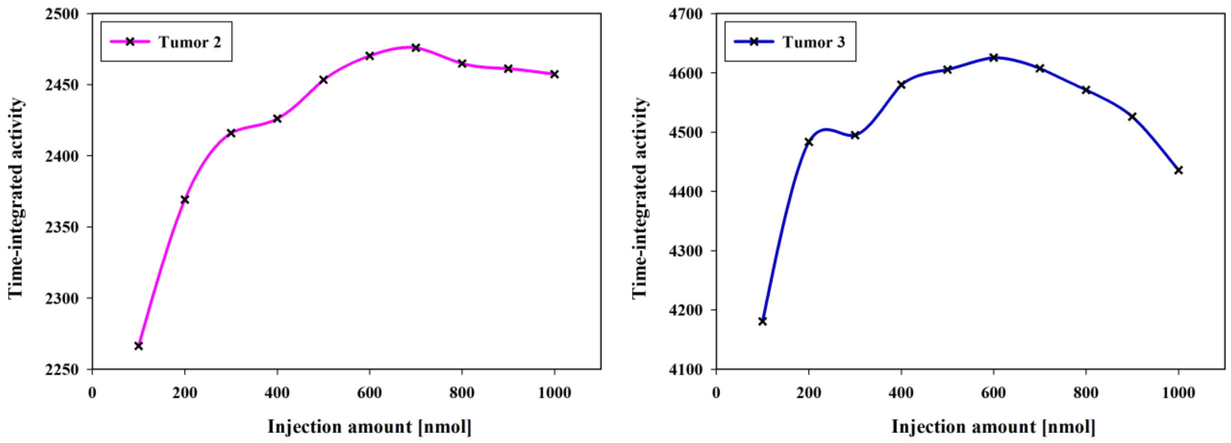

Supplementary Figure 5. Investigating the effects of the administered radiopharmaceutical amount on TIA (For tumor2 and 3). Increasing the injected quantity of labeled and unlabeled ligands initially enhances TIA, but at larger amount, receptor saturation caused by unlabeled ligands results in a reduction in TIA.

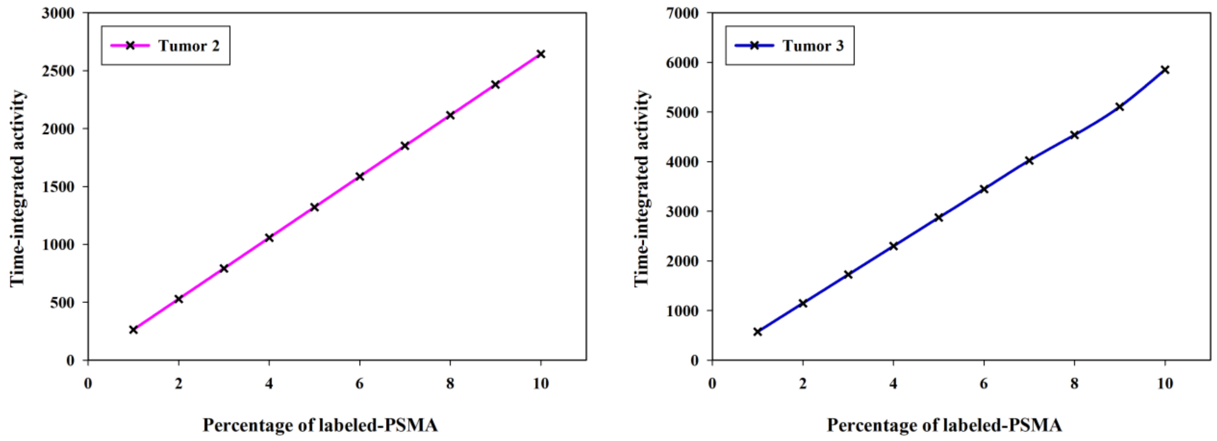

Supplementary Figure 6. Exploring the impact of the labeled PSMA-617 percentage on TIA in the tumor. The findings illustrate a notable rise in TIA with an increase in the percentage of labeled PSMA from 1% to 10%.
